# Author Correction: Localized nuclear reaction breaks boron drug capsules loaded with immune adjuvants for cancer immunotherapy

**DOI:** 10.1038/s41467-023-40690-3

**Published:** 2023-09-04

**Authors:** Yaxin Shi, Zhibin Guo, Qiang Fu, Xinyuan Shen, Zhongming Zhang, Wenjia Sun, Jinqiang Wang, Junliang Sun, Zizhu Zhang, Tong Liu, Zhen Gu, Zhibo Liu

**Affiliations:** 1 Beijing National Laboratory for Molecular Sciences, Radiochemistry and Radiation Chemistry Key Laboratory of Fundamental Science, Key Laboratory of Bioorganic Chemistry and Molecular Engineering of Ministry of Education, College of Chemistry and Molecular Engineering, Peking University, Beijing, 100871 China; 2 The Centre of Nanoscale Science and Technology and Key Laboratory of Functional Polymer Materials, Institute of Polymer Chemistry, College of Chemistry, Nankai University, Tianjin, 300071 China; 3 Key Laboratory of Advanced Drug Delivery Systems of Zhejiang Province, College of Pharmaceutical Sciences, Zhejiang University, Hangzhou, 310058 China; 4 Engineering Department, Lancaster University, Lancaster, Lancashire LA1 4YW UK; 5 College of Chemistry and Molecular Engineering, Beijing National Laboratory for Molecular Sciences, Peking University, Beijing, 100871 China; 6 Beijing Nuclear Industry Hospital, Beijing, 100045 China; 7 Beijing Capture Tech Co. Ltd, Beijing, 102413 China; 8 Jinhua Institute of Zhejiang University, Jinhua, 321299 China; 9 Department of General Surgery, Sir Run Run Shaw Hospital, School of Medicine, Zhejiang University, Hangzhou, 310016 China; 10 Liangzhu Laboratory, Zhejiang University Medical Center, 311121 Hangzhou, China; 11 Peking-Tsinghua Center for Life Sciences, Peking University, 100871 Beijing, China; 12 Changping Laboratory, 102206 Beijing, China; 13 Key Laboratory of Carcinogenesis and Translational Research (Ministry of Education/Beijing), NMPA Key Laboratory for Research and Evaluation of Radiopharmaceuticals (National Medical Products Administration), Department of Nuclear Medicine, Peking University Cancer Hospital & Institute, 100142 Beijing, China

**Keywords:** Drug delivery, Radiotherapy, Tumour immunology

Correction to: *Nature Communications* 10.1038/s41467-023-37253-x, published online 05 April 2023

The original version of this Article contained errors in Fig. 4. In particular, the Y-axes of original Fig. 4E and 4G were mislabelled. Figure 4 has now been corrected and replaced in both the PDF and HTML versions of the Article.

In addition, inaccuracies have been found in the original Source data for Figs. 2F, 2G, 4D, 4E, 4F, 4G and for Supplementary Fig. 18A and 18B. The original Source data file has now been replaced and an updated version has been provided.

These changes do not affect the scientific conclusions of the study.

