# Supplementary material for: Localized nuclear reaction breaks boron drug capsules loaded with immune adjuvants for cancer immunotherapy
Source: Nat Commun. 2023 Apr 5;14:1884. doi: 10.1038/s41467-023-37253-x (PMC10076324; doi:10.1038/s41467-023-37253-x)
Supplement: Supplementary file 1 — Supplementary Information [file 41467_2023_37253_MOESM1_ESM.pdf]

Supplementary Information for

Localized Nuclear Reaction Breaks Boron Drug Capsules Loaded with Immune Adjuvants for Cancer Immunotherapy

Yaxin Shi[1,14], Zhibin Guo[1,14], Qiang Fu[2,14], Xinyuan Shen[3], Zhongming Zhang[4], Wenjia Sun[5], Jinqiang Wang[3], Junliang Sun[5], Zizhu Zhang[6], Tong Liu[7], Zhen Gu[3,8,9,10], Zhibo Liu[1,11,12,13*]*

[1]Beijing National Laboratory for Molecular Sciences, Radiochemistry and Radiation Chemistry Key Laboratory of Fundamental Science, Key Laboratory of Bioorganic Chemistry and Molecular Engineering of Ministry of Education, College of Chemistry and Molecular Engineering, Peking University, Beijing 100871, China

[2]The Centre of Nanoscale Science and Technology and Key Laboratory of Functional Polymer Materials, Institute of Polymer Chemistry, College of Chemistry, Nankai University, Tianjin 300071, China

[3]Key Laboratory of Advanced Drug Delivery Systems of Zhejiang Province, College of Pharmaceutical Sciences, Zhejiang University, Hangzhou 310058, China;

[4]Engineering Department, Lancaster University, Lancaster, Lancashire, LA1 4YW, United Kingdom

[5]College of Chemistry and Molecular Engineering, Beijing National Laboratory for Molecular Sciences, Peking University, Beijing 100871, China

[6]Beijing Nuclear Industry Hospital, Beijing 100045, China

[7]Beijing Capture Tech Co. Ltd., Beijing 102413, China

[8]Jinhua Institute of Zhejiang University, Jinhua 321299, China

[9]Department of General Surgery, Sir Run Run Shaw Hospital, School of Medicine, Zhejiang University, Hangzhou 310016, China

[10]Liangzhu Laboratory, Zhejiang University Medical Center, Hangzhou 311121, China

[11]Peking-Tsinghua Center for Life Sciences, Peking University, Beijing 100871, China

[12]Changping Laboratory, Beijing 102206, China

[13]Key Laboratory of Carcinogenesis and Translational Research (Ministry of Education/Beijing), NMPA Key Laboratory for Research and Evaluation of Radiopharmaceuticals (National Medical Products Administration), Department of Nuclear Medicine, Peking University Cancer Hospital & Institute, Beijing 100142, China

[14]These authors contributed equally.

*Corresponding authors, e-mails: guzhen@zju.edu.cn; zbliu@pku.edu.cn

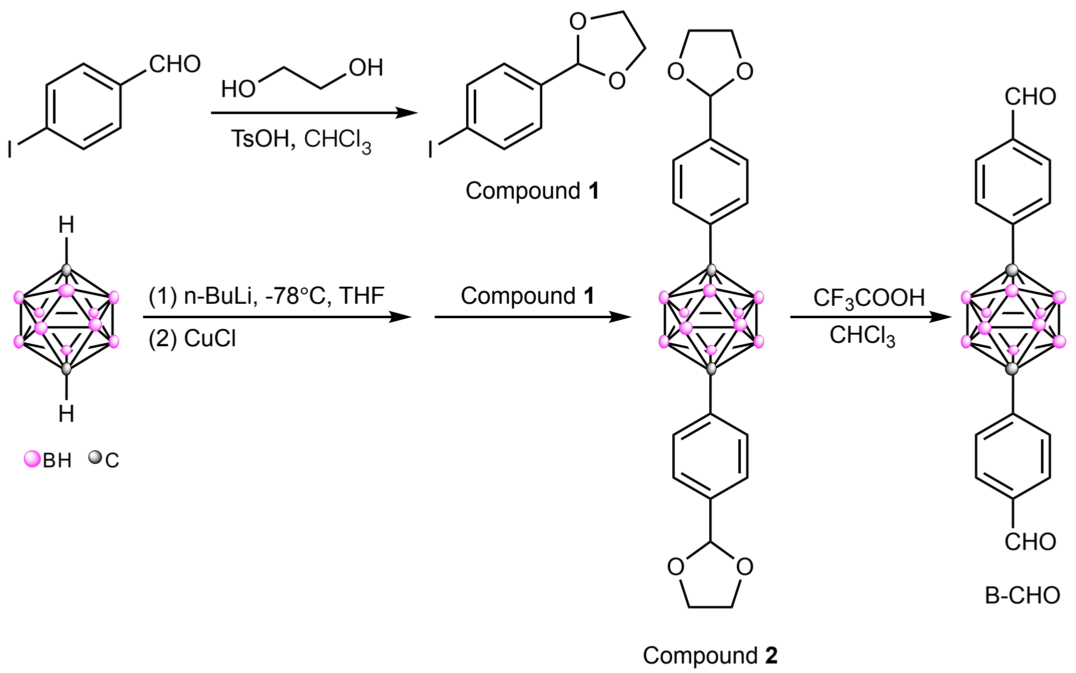

Supplementary Figure 1. Synthesis route of monomer *p*-carborane-1,10-phenyl-dialdehyde (B-CHO)

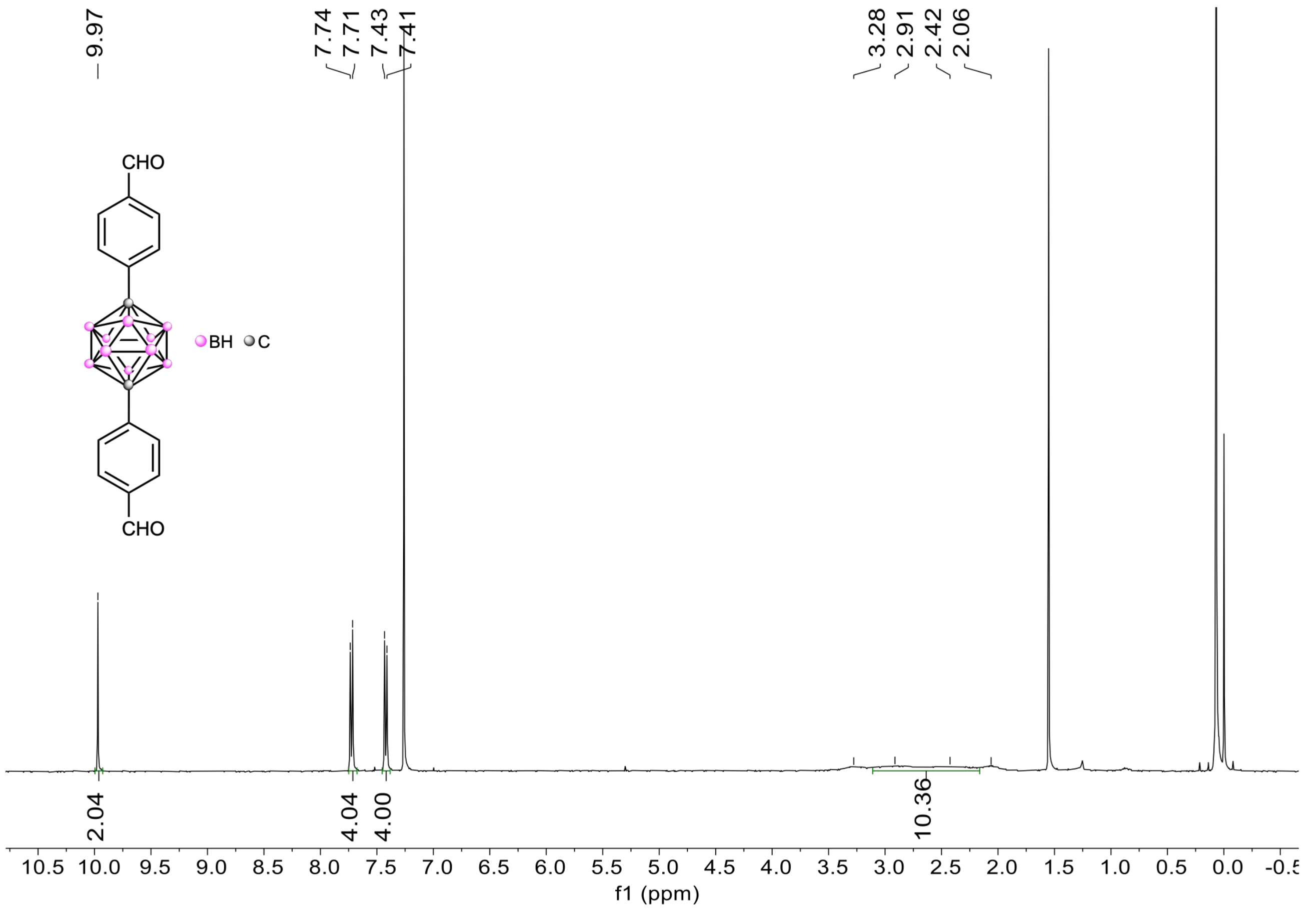

Supplementary Figure 2. ^{1}**H NMR spectrum of B-CHO in** $CDCl_3$**.**

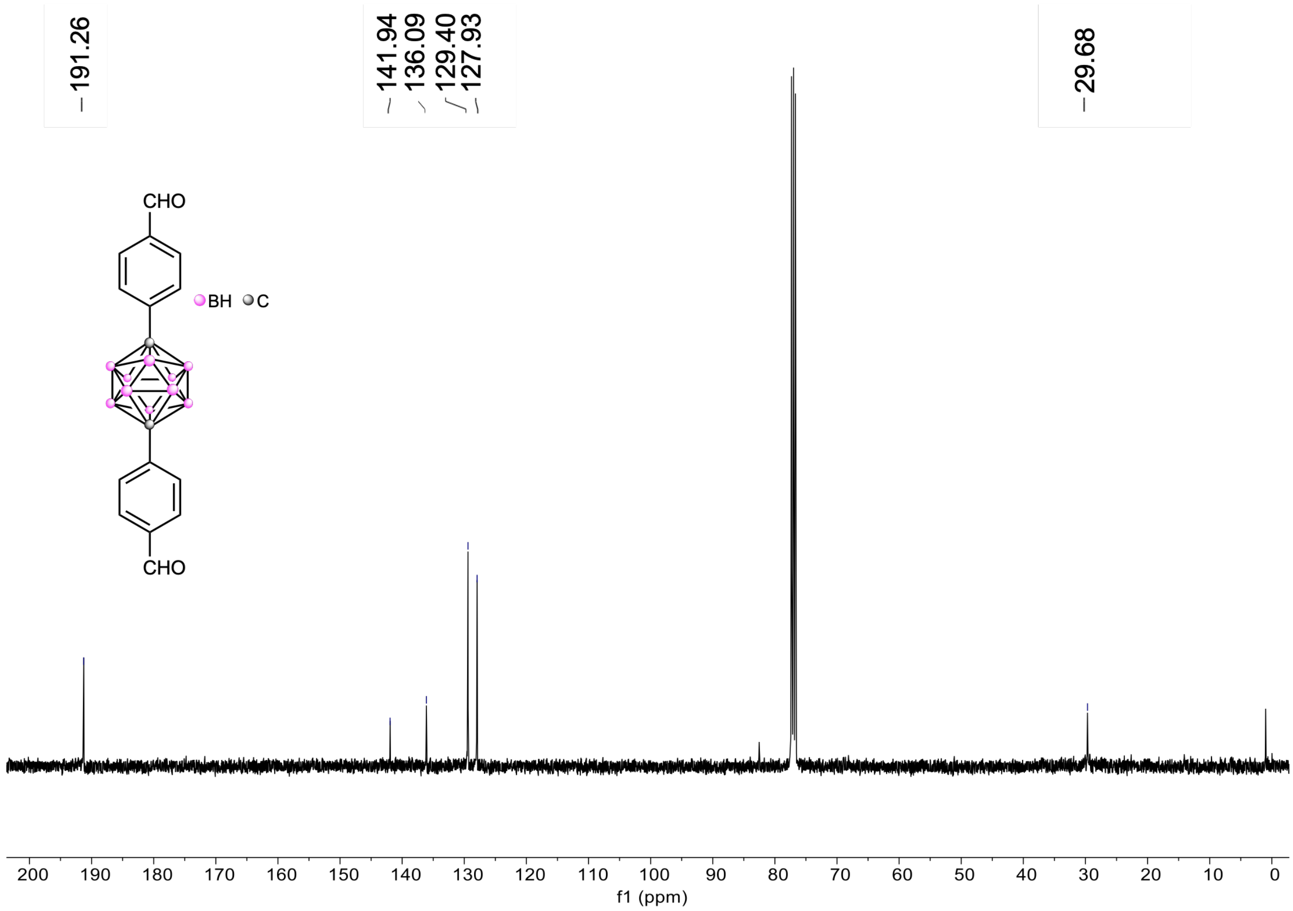

Supplementary Figure 3. ^{13}**C NMR spectrum of B-CHO in CDCl$_3$.**

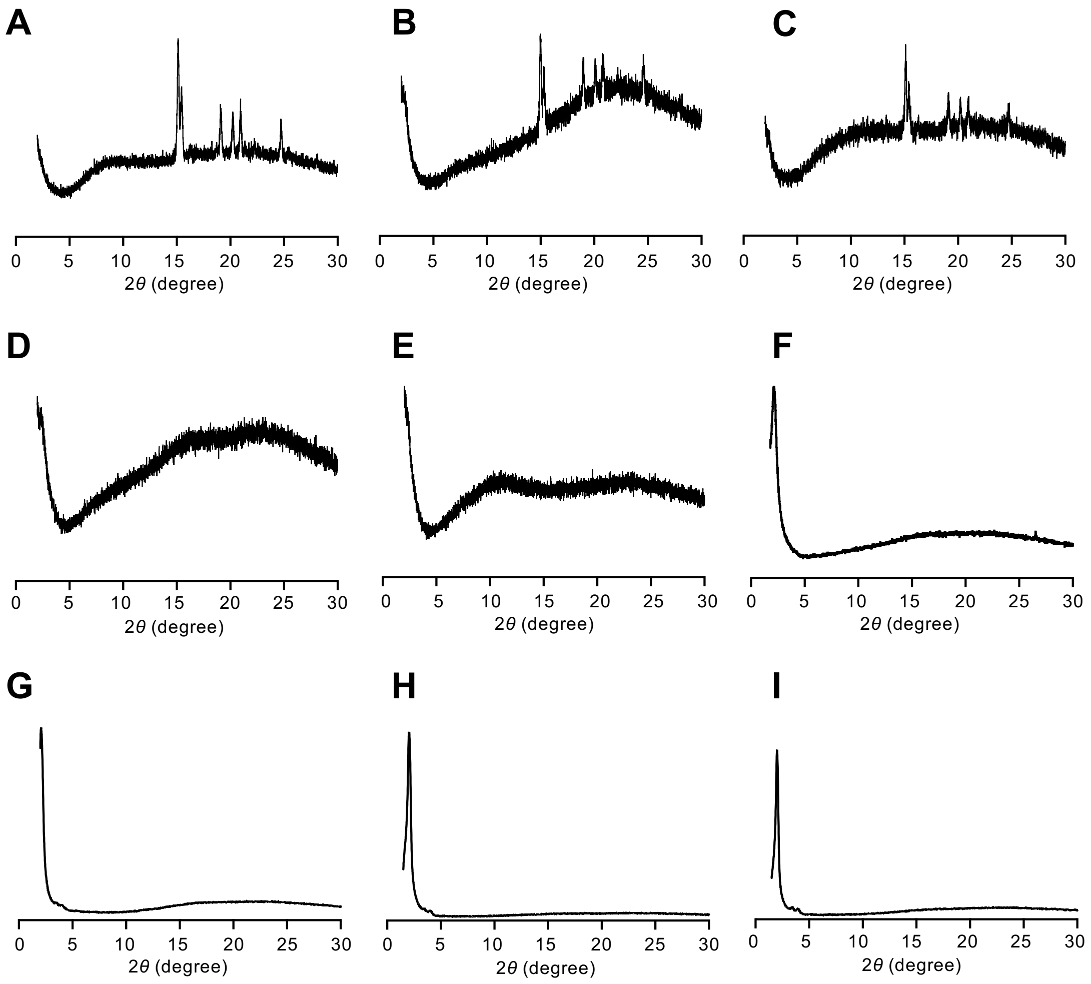

Supplementary Figure 4. Powder X-ray diffraction (PXRD) of B-COF synthesized in different conditions.

(**A**), Methanol. (**B**), Ethanol. (**C**), *n*-butanol. (**D**), Acetone. (**E**), Mesitylene. (**F**), *o*-dichlorobenzene. (**G**), *o*-dichlorobenzene, 37 °C. (**H**), *o*-dichlorobenzene, 50 °C. (**I**), *o*-dichlorobenzene, 50 °C with polystyrene microspheres. In brief, the organic linkers of TAPB and B-CHO in the molar ratio of 2:3 were added to different solvents (2.5 mL) containing HAc (0.15 mL, 12 M).

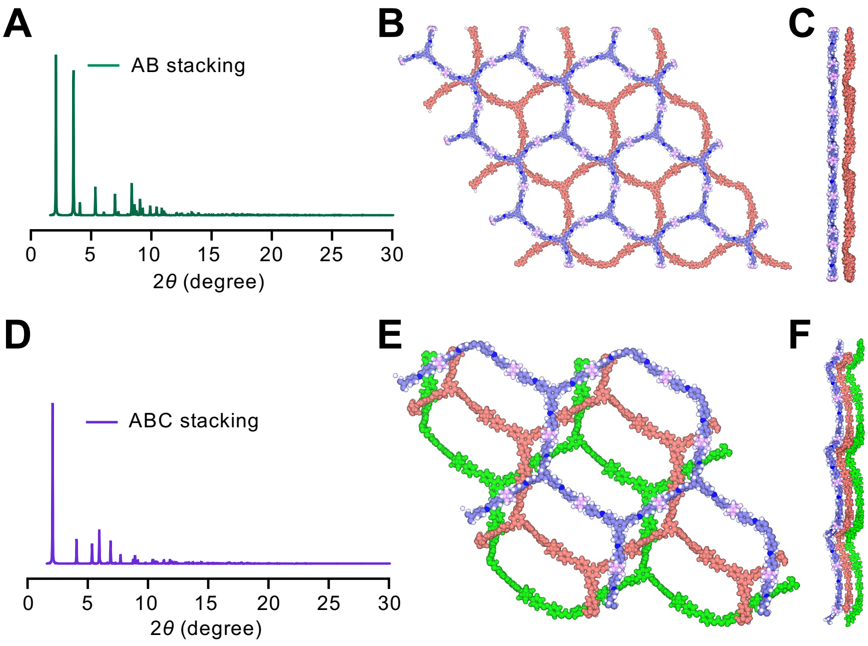

Supplementary Figure 5. Simulated PXRD patterns and the staggered structure models (AB and ABC) of B-COF.

(**A**, **D**), PXRD patterns of AB (**A**) and ABC (**D**) stacking mode. (**B**, **C**, **E**, **F**), The reconstructed structure of the AB (**B**, **C**) and ABC (**E**, **F**) stacking mode of B-COF at the top view and side view, respectively.

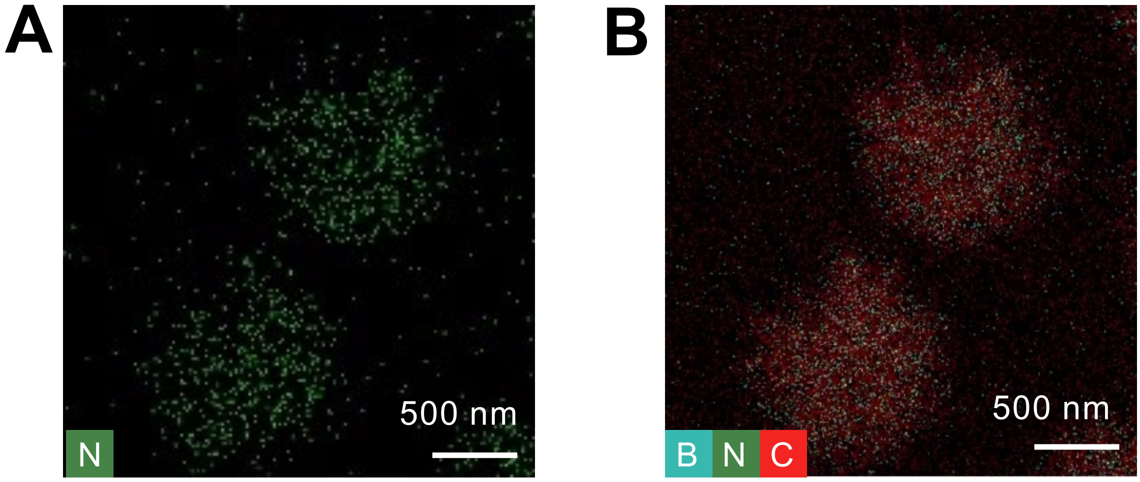

Supplementary Figure 6. The corresponding energy dispersive spectroscopy (EDS) elemental mapping images of B-COF in Figure 1G.

(**A**), The elemental mapping of N in B-COF (N as green, $n = 3$ samples). (**B**), The elemental mapping of B-COF (C, B, N, merge, $n = 3$ samples).

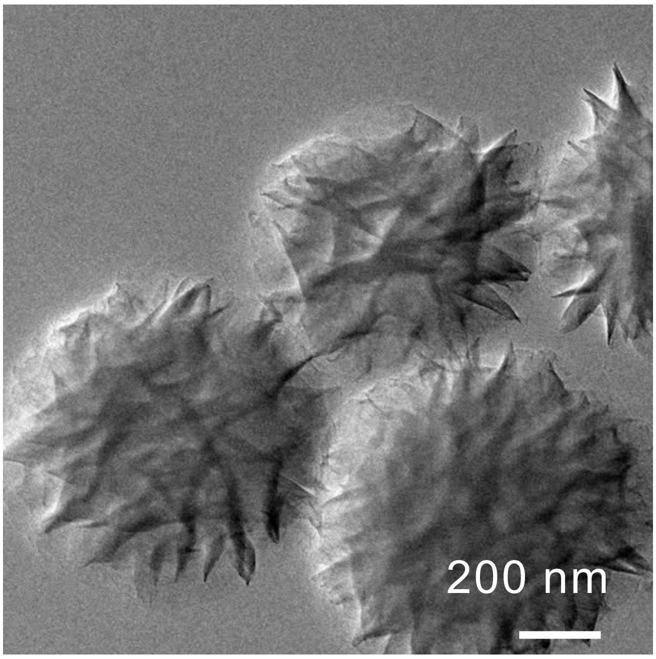

Supplementary Figure 7. TEM imaging of imiquimod loaded B-COF. The experiment was repeated three times independently (n = 3 samples) with similar results.

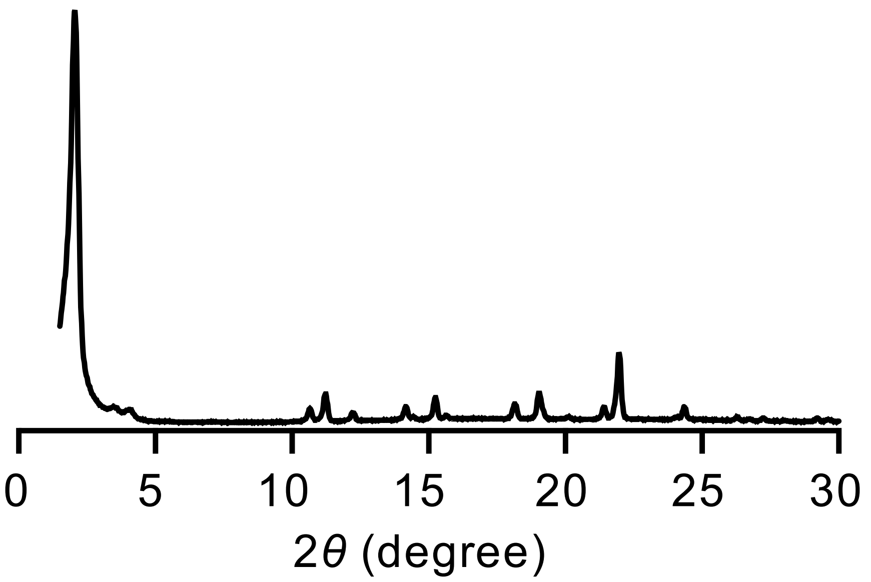

Supplementary Figure 8. PXRD of imiquimod loaded B-COF.

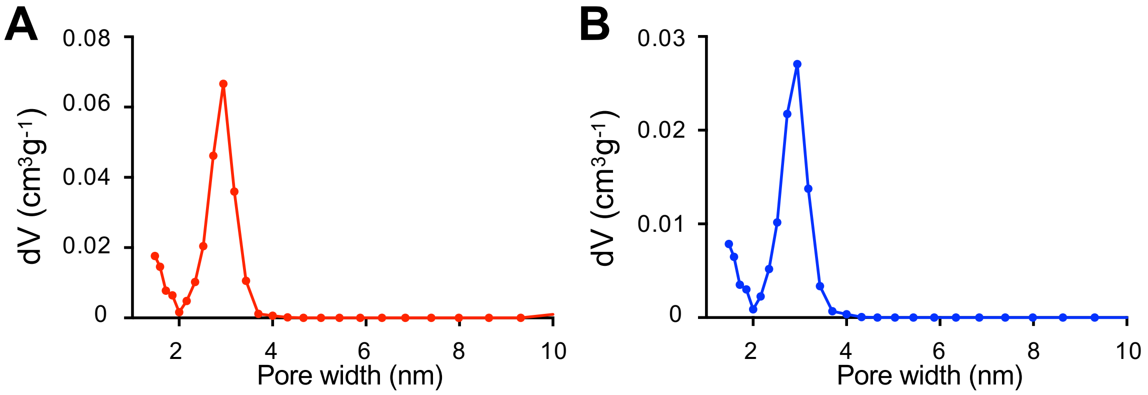

Supplementary Figure 9. Pore size distributions from fitting the nonlocal density functional theory (NLDFT) model to the adsorption data of B-COF (A) and imiquimod loaded B-COF (B).

The Brunauer-Emmett-Teller (BET) surface area of B-COF is 673 m^2 g^{-1}, with a total pore volume of 0.68 cm^3 g^{-1} (pore size, 2.95 nm). They were reduced to 275 m^2 g^{-1} and 0.31 cm^3 g^{-1} (pore size, 2.95 nm) after drug loading (*n* = 3 samples), respectively.

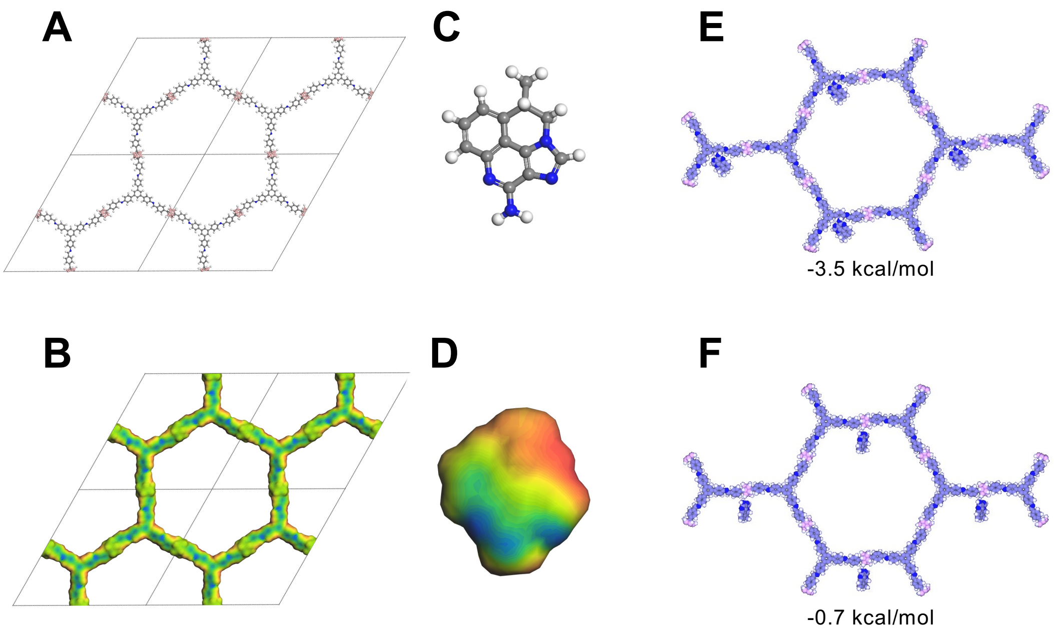

Supplementary Figure 10. Theoretical simulation helps to further understand the binding site of payload in boron capsule.

(**A**–**D**), Molecular structure and electrostatic potential map of (**A**, **B**) B-COF and (**C**, **D**) imiquimod. (**E**, **F**), Theoretical model and binding energy calculated by density functional theory (DFT) when imiquimod is adsorbed near the benzene ring unit (**E**) and the carborane unit (**F**), respectively.

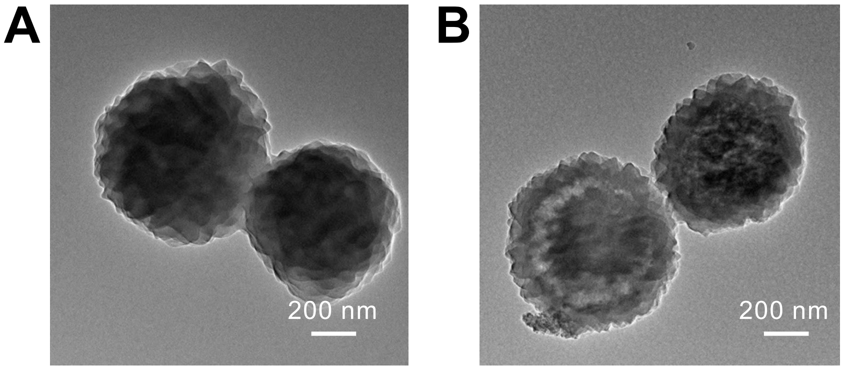

Supplementary Figure 11. TEM imaging of boron capsule (A) and B-COF (B). The experiments for A and B were repeated three times independently (n = 3 samples) with similar results.

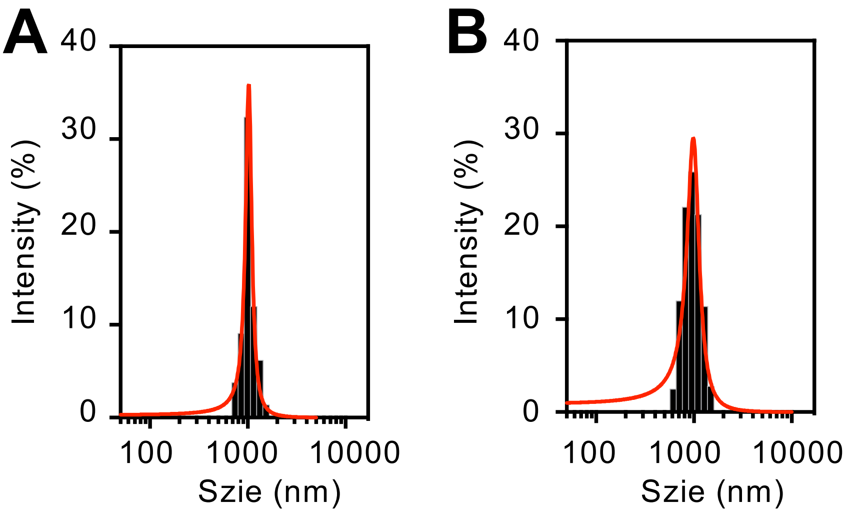

Supplementary Figure 12. The hydrodynamic size of boron capsule in PBS (A) and serum (B) at 37 °C for 7 days (*n* = 3 samples).

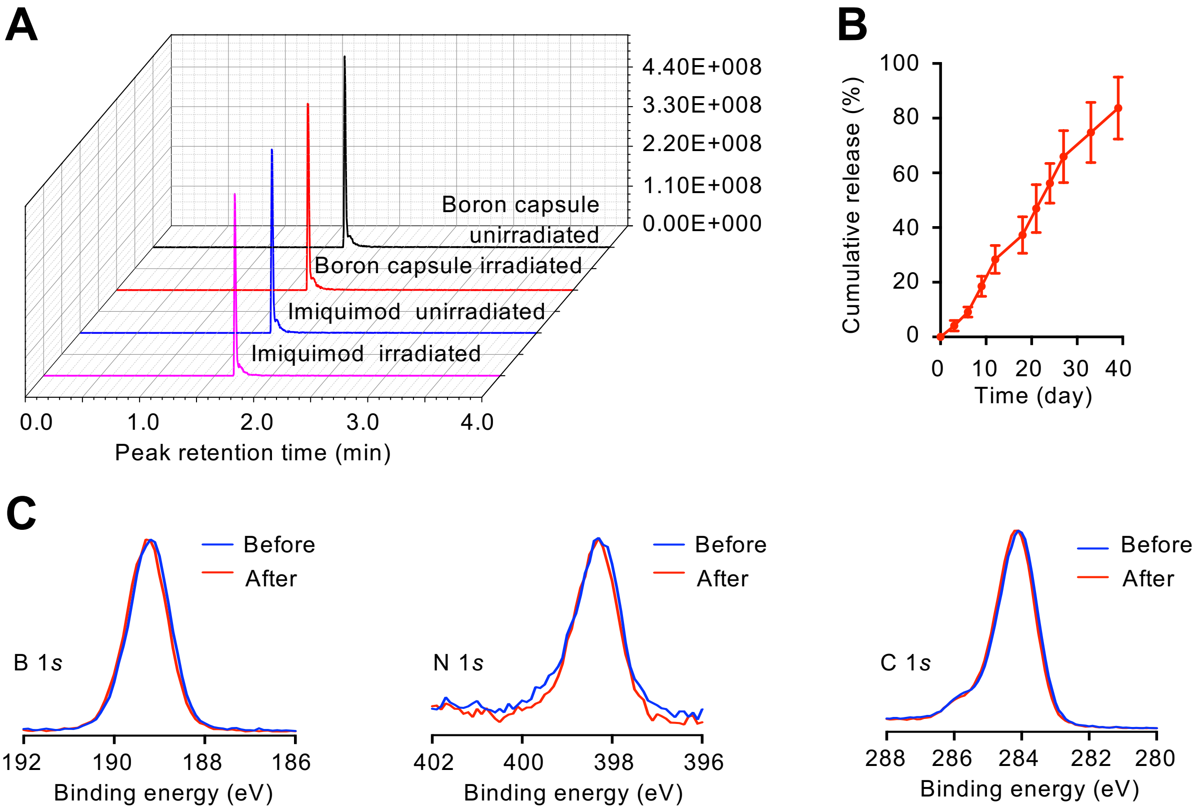

Supplementary Figure 13. Boron neutron capture reaction affects neither the stability of imiquimod nor the major structure of boron capsule.

(**A**), UPLC-MS analysis of imiquimod under the different condition with and without neutron irradiation. (**B**), Cumulative release profile of boron capsule after neutron irradiation. Data shown as mean ± s.d. ($n = 3$ independent experiments). (**C**), X-ray photoelectron spectroscopy (XPS) spectrum shows that the chemical state of boron, nitrogen and carbon element in B-COF has no significant change before (blue) and after (red) neutron irradiation, respectively.

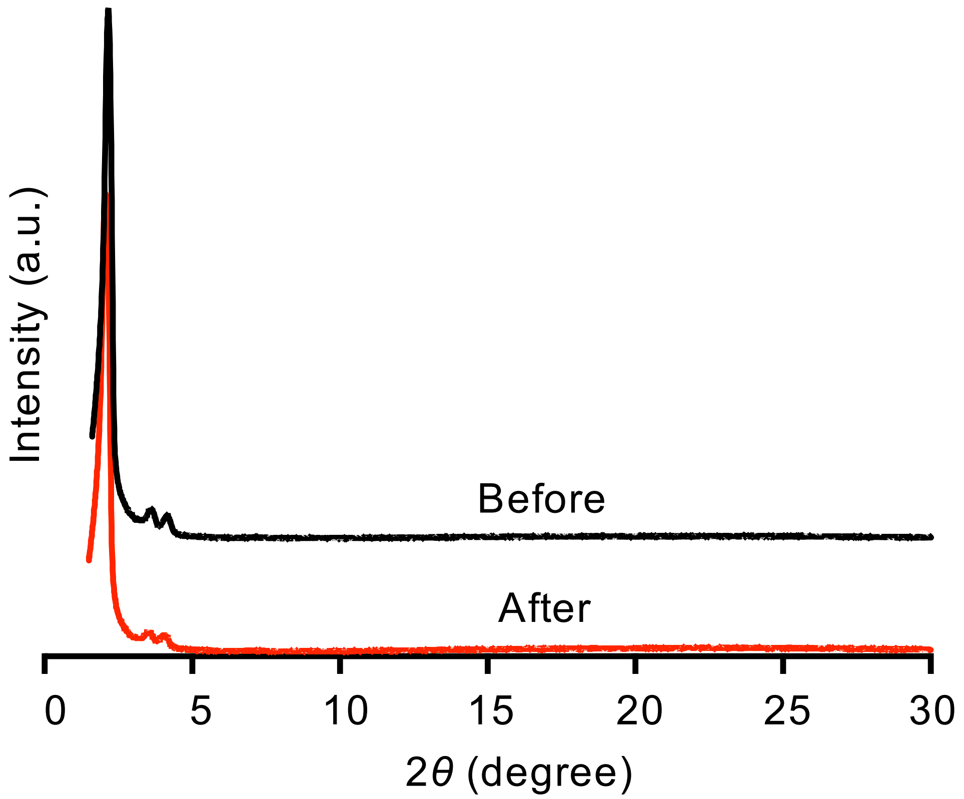

Supplementary Figure 14. PXRD of B-COF before and after neutron irradiation.

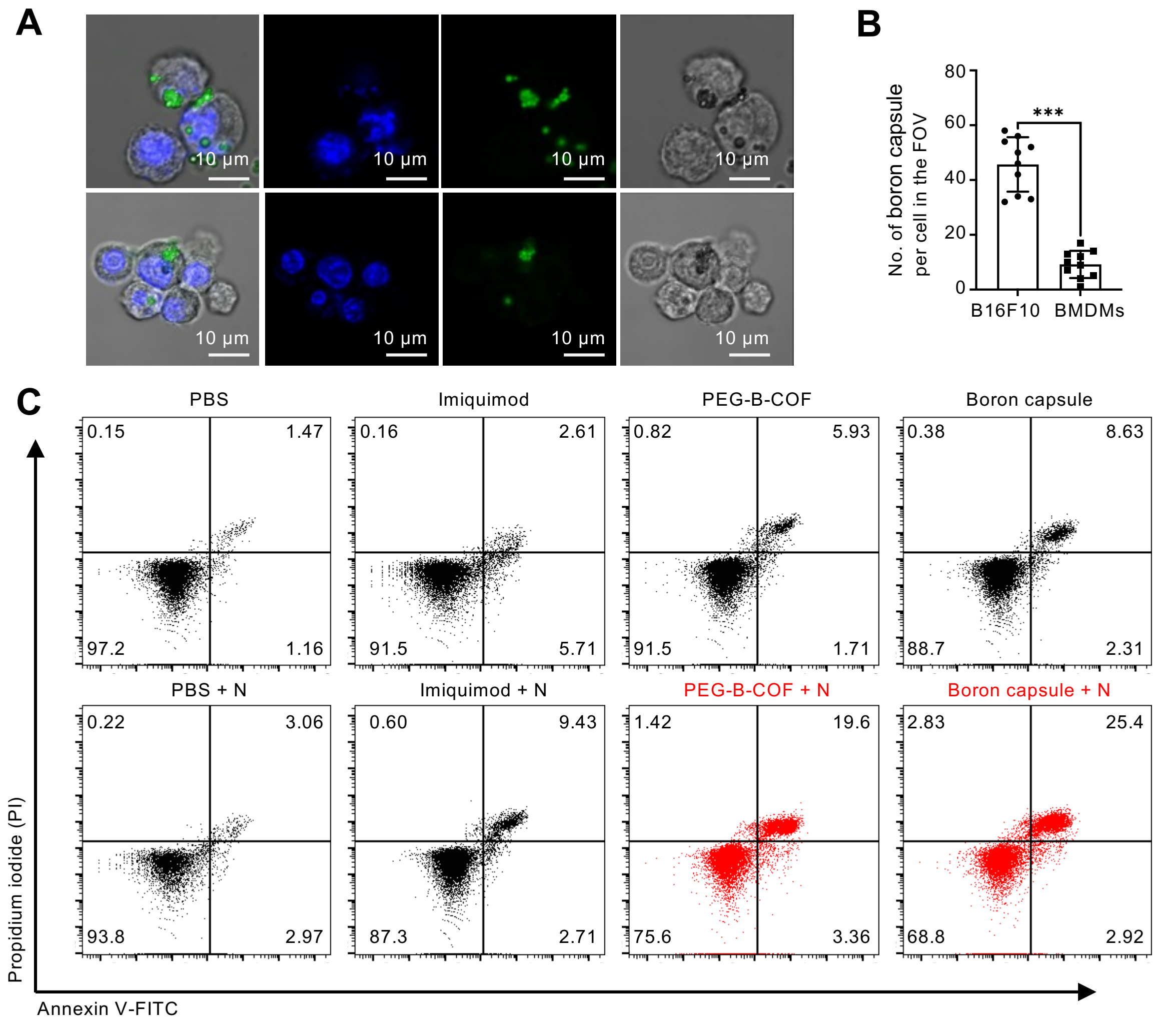

Supplementary Figure 15. Primary mouse bone-marrow-derived macrophages (BMDMs) is not as sensitive as B16F10 in cellular uptake of boron capsule and related cell death.

(A), Representative confocal images of BMDMs cells are shown after being treated by FITC fluorophore-conjugated boron capsule (1 mg/mL) for 2 hours (n = 3 samples). (B), Number of boron capsule per cell in the giving field of view (FOV) by confocal microscopy. Data are shown as mean ± s.d. (n = 10 fields of view). (C), The cell death rate was calculated as Annexin V–FITC+/PI+ cells and Annexin V–FITC+/PI− cells (n = 3 samples).

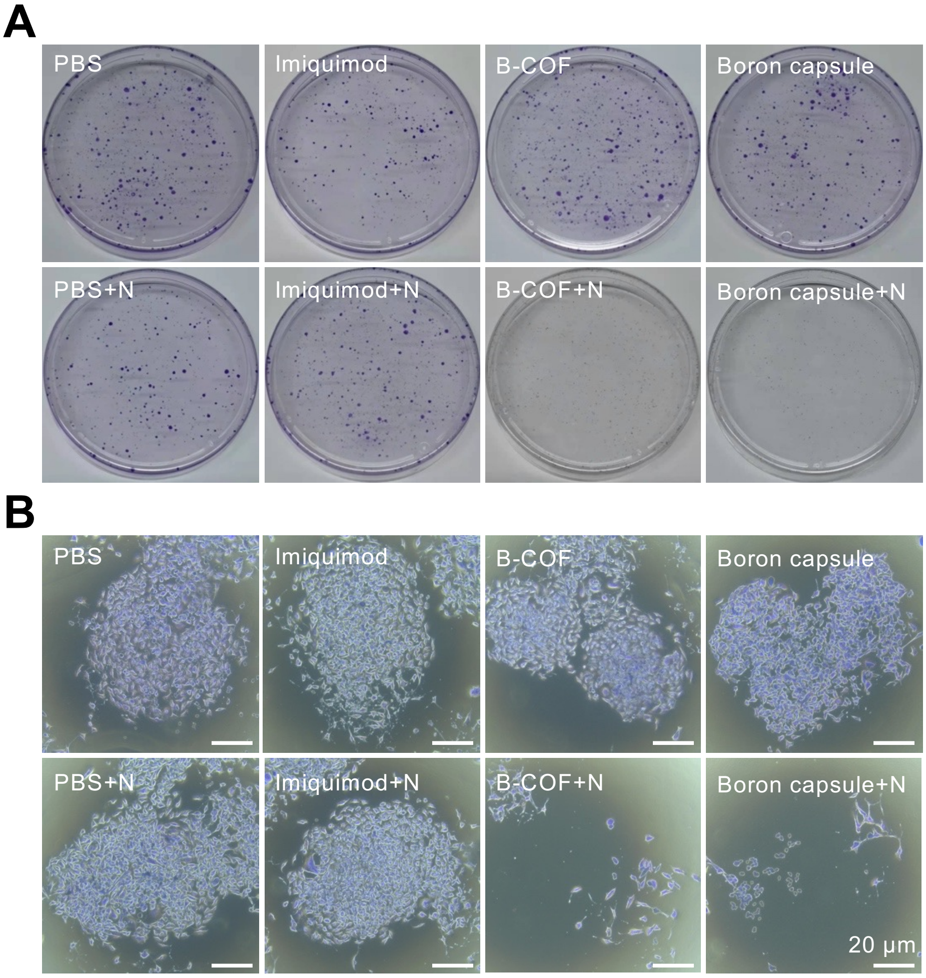

Supplementary Figure 16. Boron neutron capture reaction remarkably inhibits the growth of cancer cells.

Representative images of total colonies in a 10 cm Petri dish (**A**) and a representative cell colony (**B**) of B16F10 cells with indicated treatments (*n* = 4). Scale bars, 20 µm.

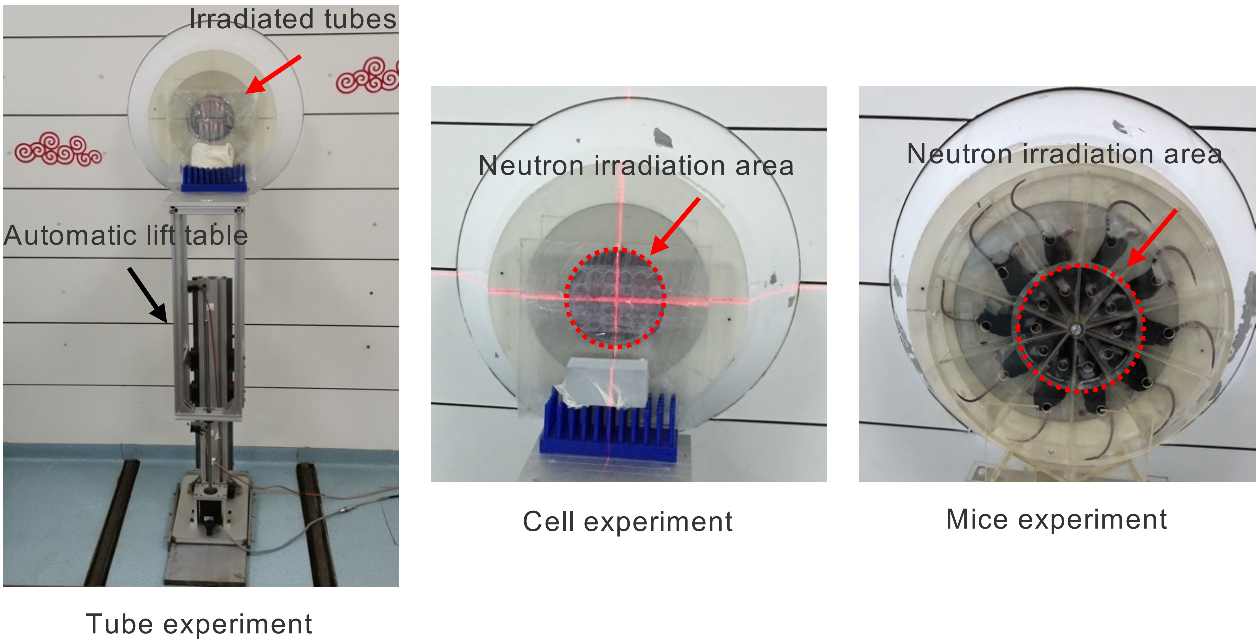

Supplementary Figure 17. The photos of the thermal neutron beam facility for neutron-irradiation experiments of tubes, cells and mice.

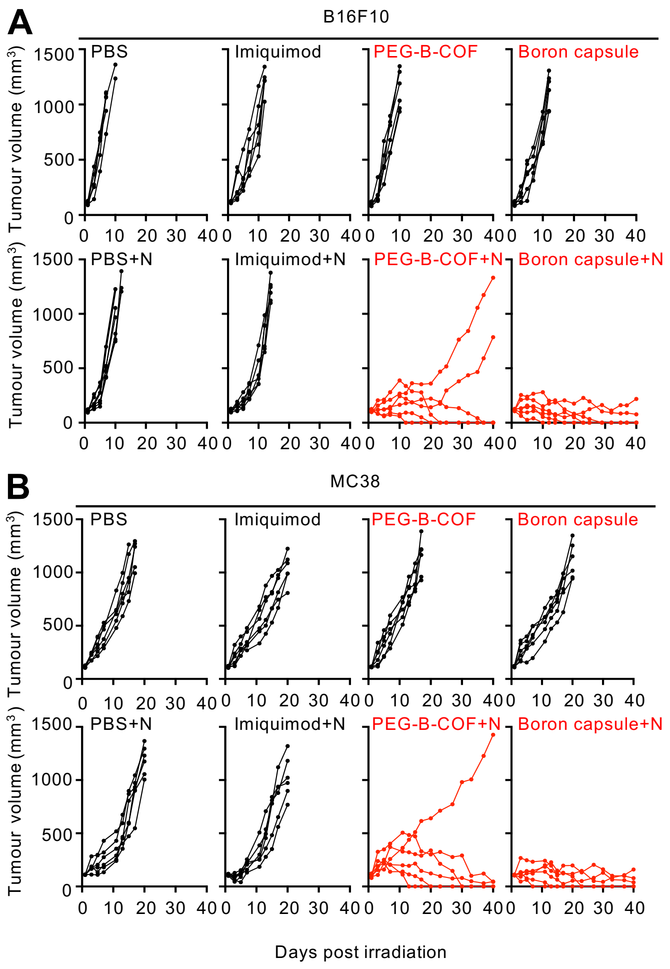

Supplementary Figure 18. B16F10 (A) and MC38 (B) Tumour volume of the individual mouse at indicated time points after implantation.

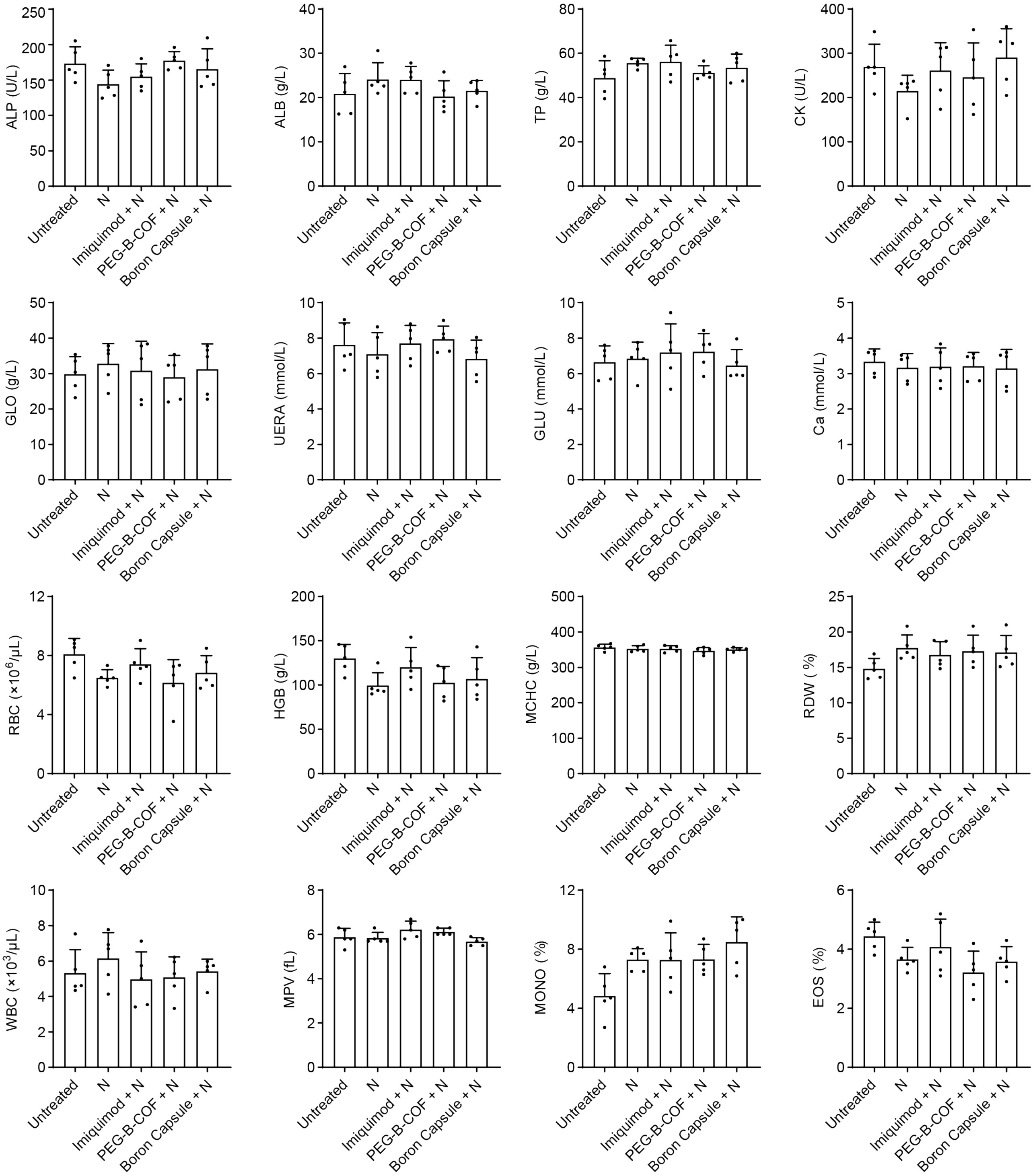

Supplementary Figure 19. The blood routine and biochemical test of B16F10 tumour-bearing mice treated with Neurtron (N) alone, Imiquimod, PEG-B-COF + N and Boron Capsule + N at day 14 (*n* = 4 mice). Data are means ± SD..

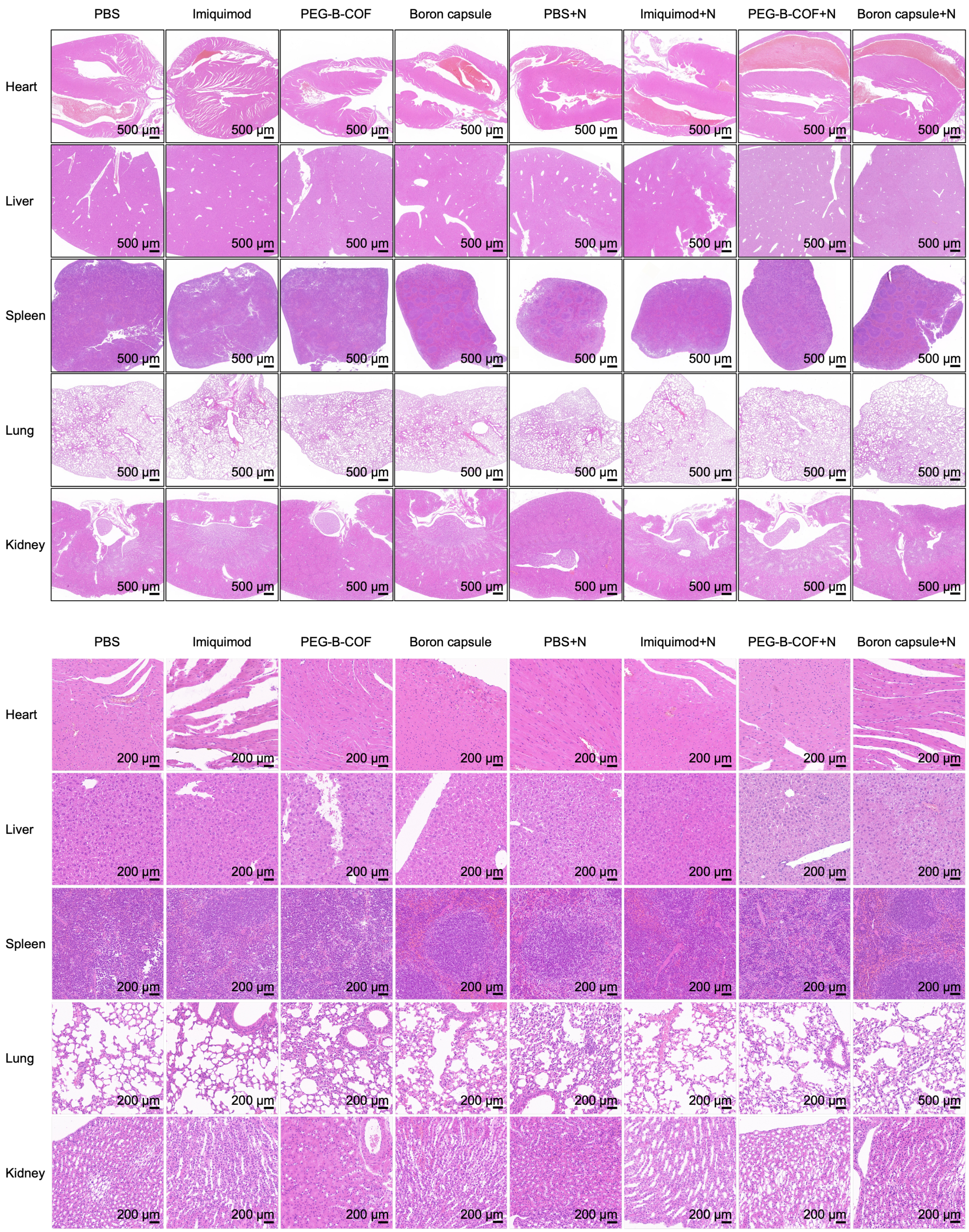

Supplementary Figure 20. Representative H&E staining of major organs with different treatments (*n* = 3 samples).

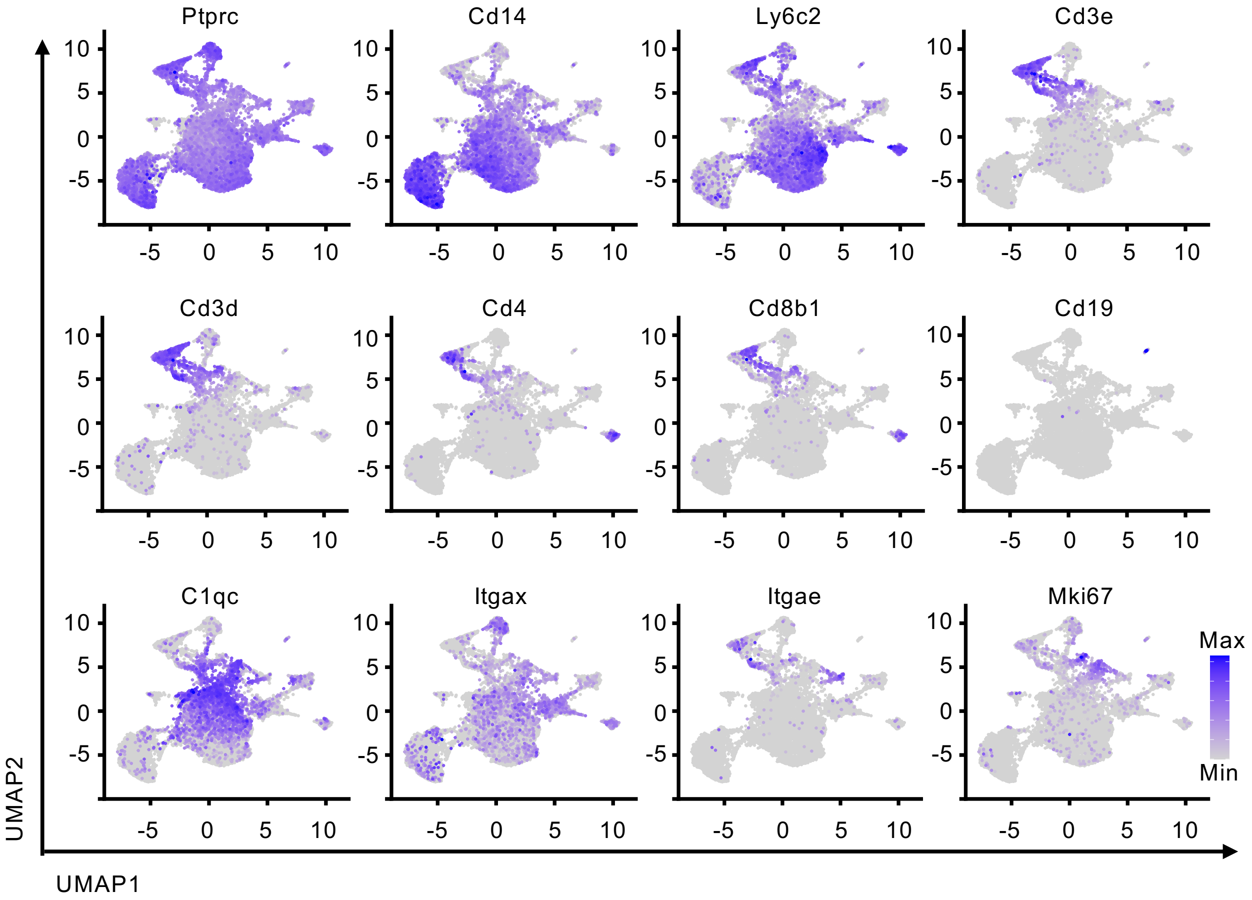

Supplementary Figure 21. Signature gene-expression patterns for the corresponding cell clusters on the UMAP plot.

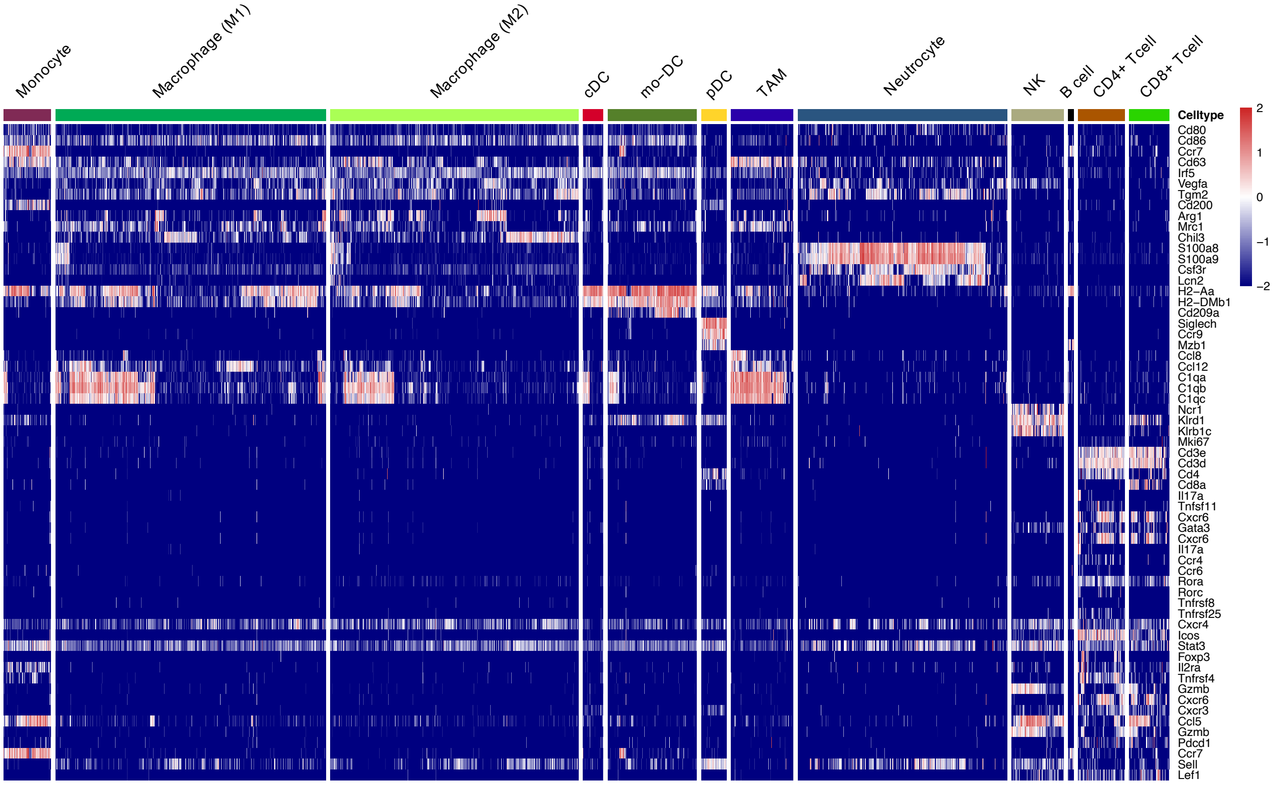

Supplementary Figure 22. Heat map of immune-cell clusters with unique signature genes.

Colors on top of the map indicate the immune-cell clusters. Conventional dendritic cell (cDC); monocyte-derived dendritic cell (mo-DC); Plasmacytoid dendritic cell (pDC); tumour-associated macrophage (TAM); natural killer cell (NK).

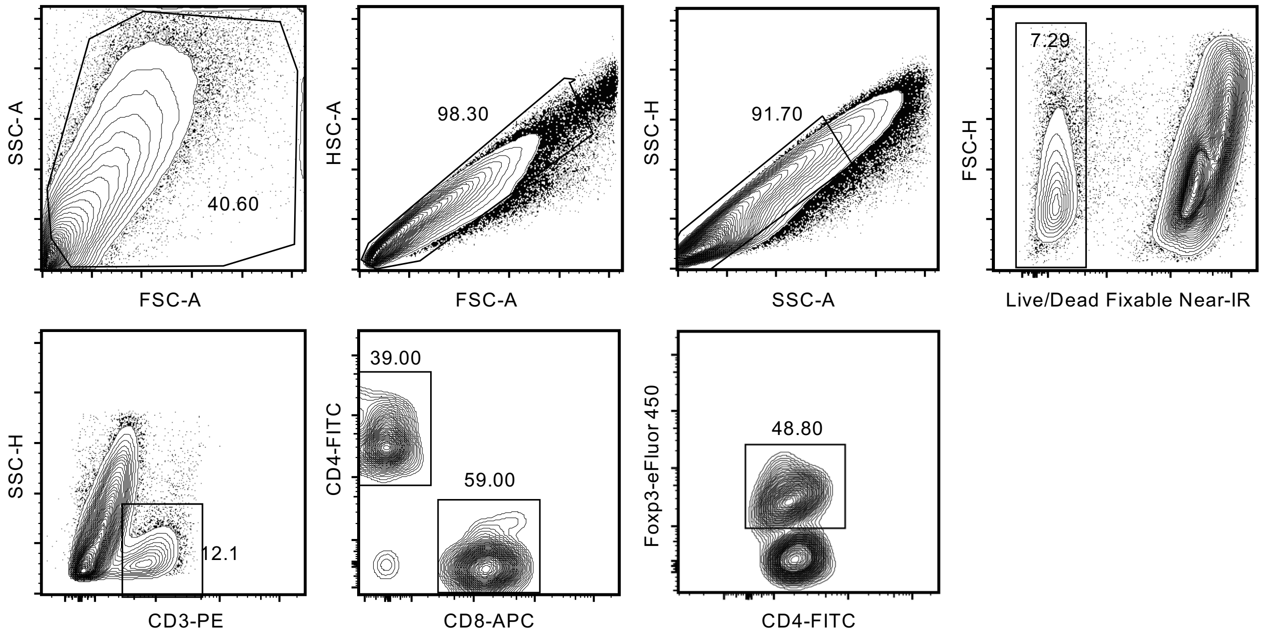

Supplementary Figure 23. Representative gating strategies for CD3+ cells, CD4+ T cells, CD8+ T cells and Foxp3+ T cells.

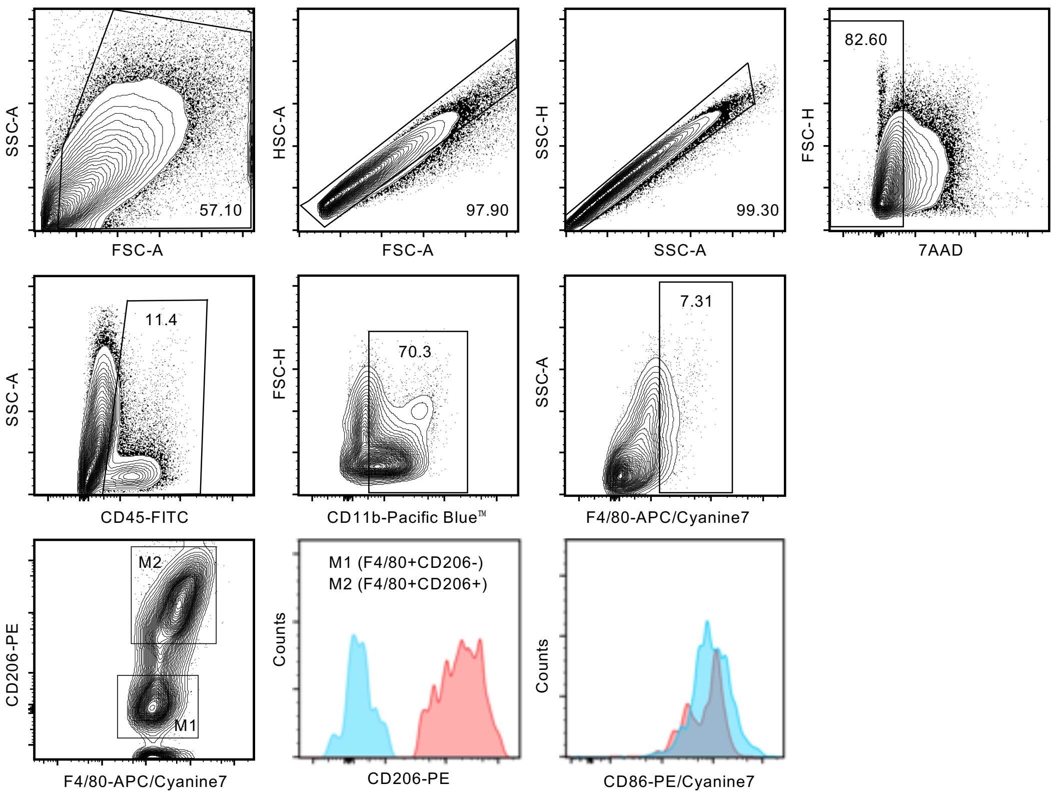

Supplementary Figure 24. Representative gating strategies for macrophages.

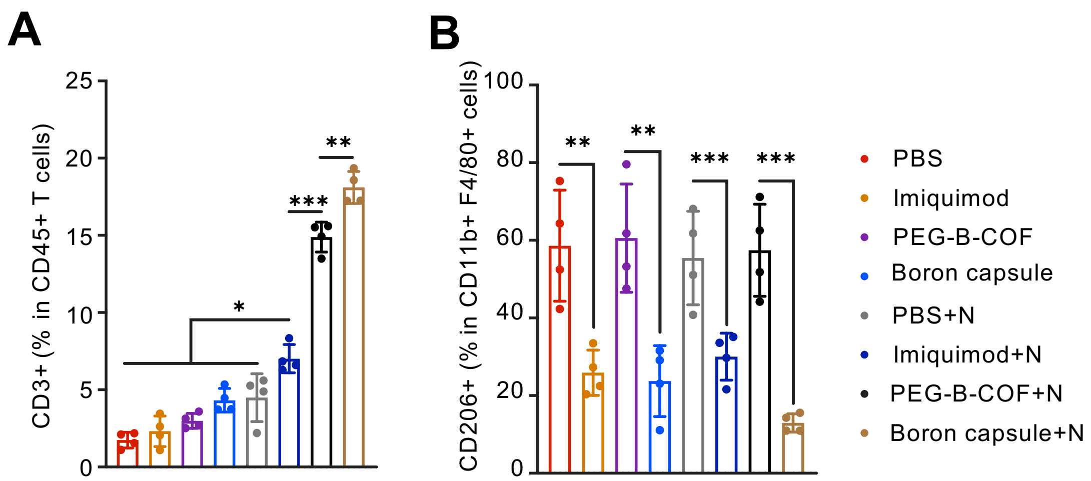

Supplementary Figure 25. BNCT with boron capsule exhibits robust immune responses *in vivo*.

(**A, B**), Percentages of CD3+ (**A**) and M2 (**B**) macrophages in B16F10 tumours. Data shown as mean ± s.d. ($n = 4$ independent experiments). One-way analysis of variance (ANOVA) followed by Tukey's HSD post hoc test. *$P < 0.05$, **$P < 0.01$, ***$P < 0.001$.

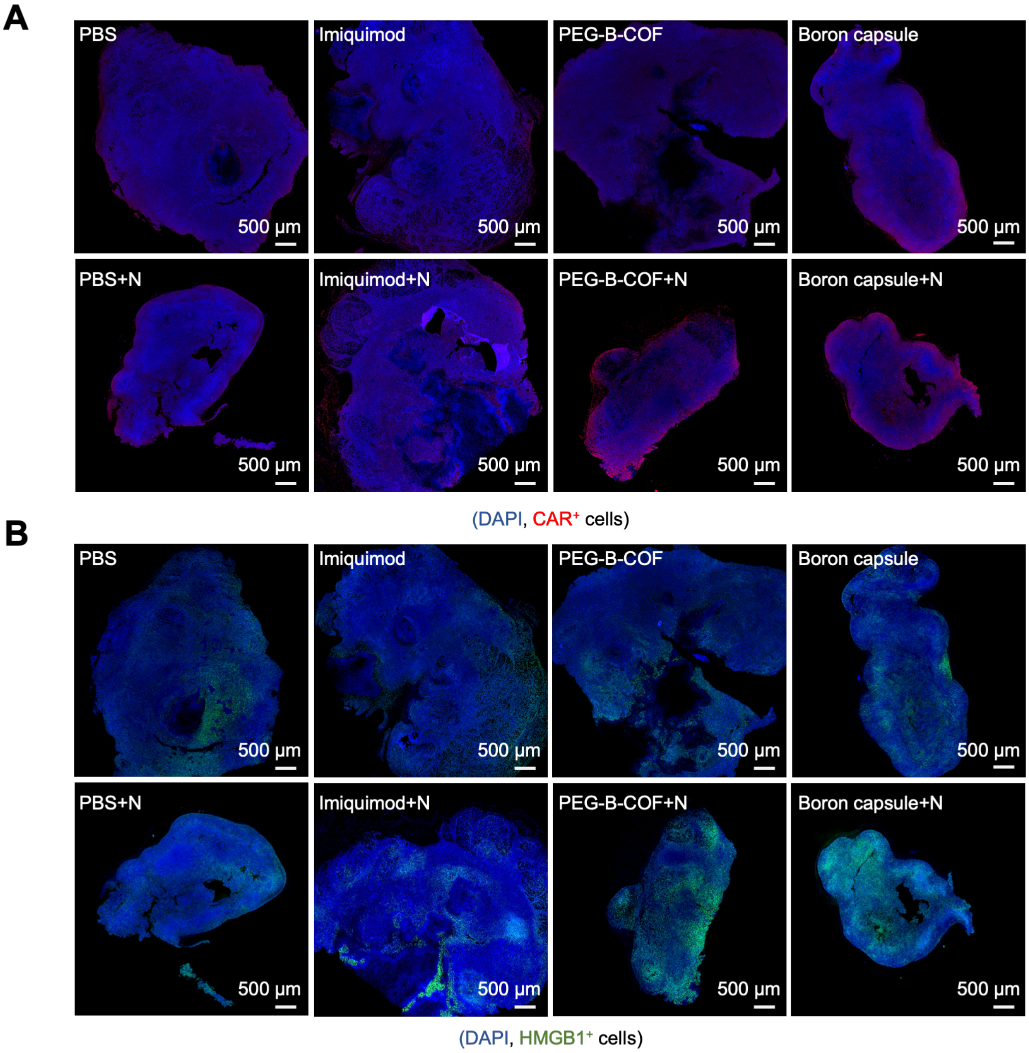

Supplementary Figure 26. BNCT with boron capsule induces the immunogenic cell death in tumour.

Representative immunofluorescence staining of calreticulin (CRT) exposure (**A**) and high-mobility group box 1 protein (HMGB1) release (**B**) are shown. Tumour were harvested 7 days after the indicated treatment.

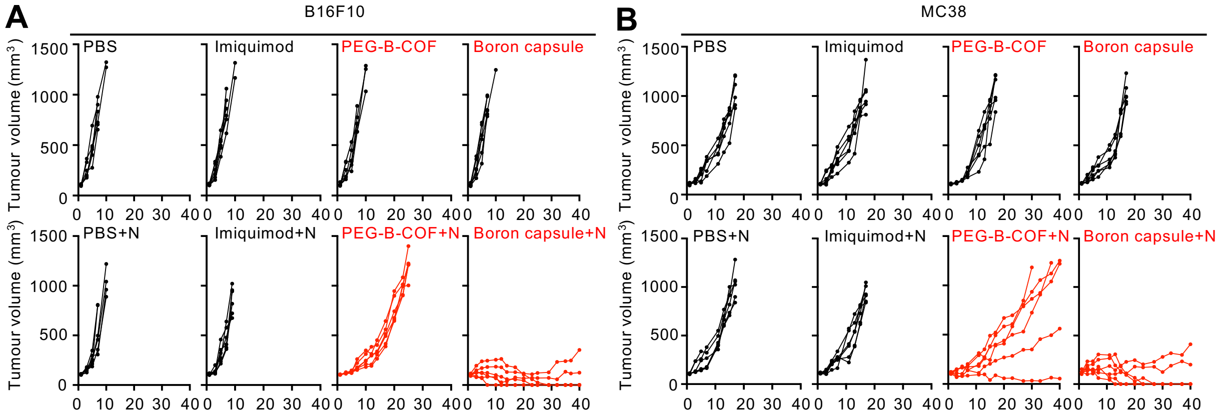

Supplementary Figure 27. The distant tumour volume of the individual mouse at indicated time points after implantation.

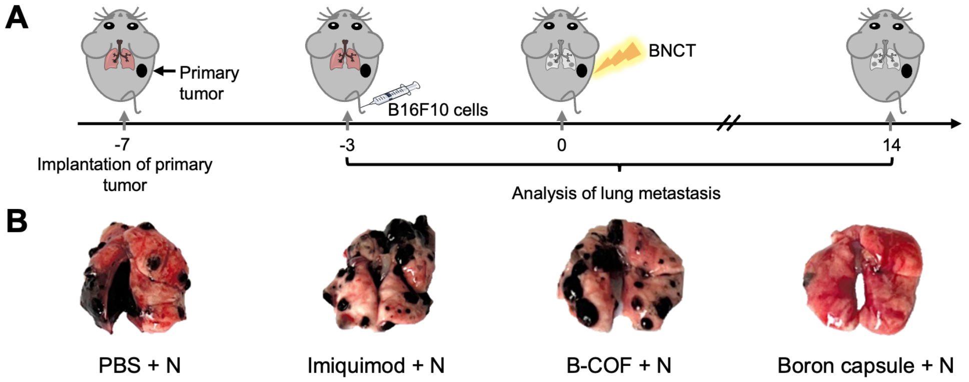

Supplementary Figure 28. Metastasis inhibition in lung metastatic B16F10 and subcutaneous B16F10 tumour models by BNCT with immunological adjuvant-loaded boron capsule.

(**A**), Treatment scheme. (**B**), Representative lung photographs for each treatment group (n = 6 mice).

Supplementary Table 1. Reaction conditions screening for B-COF synthesis.

Entry	TPB (mmol)	Solvents	Volume (mL)	Catalyst	Temp.	Time	Adding	Remarks
1	0.02	methanol	2.5	12M HAc 150 μL	R.T	8 h		poor
2	0.02	ethanol	2.5	12M HAc 150 μL	R.T	8 h		poor
3	0.02	*n*-butanol	2.5	12M HAc 150 μL	R.T	8 h		poor
4	0.02	acetone	2.5	12M HAc 150 μL	R.T	8 h		poor
5	0.02	mesitylene	2.5	12M HAc 150 μL	R.T	8 h		poor
6	0.02	*o*-dichlorobenzene	2.5	12M HAc 150 μL	R.T	8 h		poor
7	0.02	*o*-dichlorobenzene	2.5	12M HAc 150 μL	37°C	8 h		excellent
8	0.02	*o*-dichlorobenzene	2.5	12M HAc 150 μL	50°C	8 h		excellent
9	0.02	*o*-dichlorobenzene	2.5	12M HAc 150 μL	R.T	8 h	PS 50 μL	excellent

Supplementary Table 2. Atomistic coordinates for the AA-stacking mode of B-COF optimized by using the Materials Studio Forcite molecular dynamics module (Universal force fields).

B-COF: Space group symmetry *P*3							
$a = b = 51.2534$ Å, $c = 5.3349$ Å, $\alpha = \beta = 90^{o}$, $\gamma = 120^{o}$							
	X	Y	Z		X	Y	Z
N1	0.82587	0.41860	2.30183	H37	0.49729	0.52514	3.81591
C2	0.65031	0.34863	2.16461	C38	0.55681	0.40088	3.18428
C3	0.68189	0.36483	2.16323	C39	0.41129	0.57868	3.75914
C4	0.63312	0.36455	2.18879	C40	0.49217	0.46542	3.38653
C5	0.74567	0.35920	2.37586	C41	0.50851	0.44765	3.33866
C6	0.77678	0.37634	2.40912	C42	0.53511	0.45472	3.47132
C7	0.79414	0.40133	2.25648	H43	0.54401	0.47197	3.61417
C8	0.78001	0.40886	2.06626	C44	0.55078	0.43953	3.42350
C9	0.74883	0.39177	2.03353	C45	0.54033	0.41674	3.24197
N10	0.42620	0.82084	0.95589	C46	0.51360	0.40881	3.11425
C11	0.35053	0.69830	1.25192	C47	0.49763	0.42372	3.16404
C12	0.36479	0.68104	1.25571	H48	0.47686	0.41626	3.06333
C13	0.36011	0.74238	0.99020	B49	0.49817	0.49343	3.21288
C14	0.40584	0.79075	1.03898	H50	0.51774	0.50227	3.06212
C15	0.41365	0.77950	1.24758	B51	0.51101	0.49975	3.49371
C16	0.39517	0.74965	1.32432	H52	0.53736	0.51217	3.53405
C17	0.36242	0.63162	1.19454	B53	0.48651	0.47174	3.66434
C18	0.39342	0.62137	0.91282	H54	0.49571	0.46157	3.81501
C19	0.45997	0.50145	3.49157	B55	0.45791	0.44837	3.49316
C20	0.44430	0.51978	3.54300	H56	0.44477	0.42200	3.53418
C21	0.42111	0.51050	3.72315	B57	0.46464	0.46138	3.21317

H22	0.41172	0.48904	3.81652	H58	0.45391	0.44209	3.06163
C23	0.41005	0.52936	3.79353	H59	0.44992	0.58717	0.38848
C24	0.42093	0.55746	3.67824	H60	0.39321	0.52206	0.94009
C25	0.44188	0.56584	3.48405	H61	0.57117	0.44577	0.52833
C26	0.45347	0.54730	3.41702	H62	0.50500	0.39096	-0.02429
H27	0.47003	0.55496	3.26863	H63	0.62958	0.41697	0.55671
B28	0.45404	0.47323	3.66479	H64	0.60206	0.34033	-0.11282
H29	0.43474	0.46350	3.81656	H65	0.57205	0.36501	-0.05568
B30	0.44103	0.46726	3.38477	H66	0.65962	0.39224	0.50035
H31	0.41472	0.45564	3.34381	H67	0.69368	0.38924	0.16972
B32	0.46563	0.49514	3.21372	H68	0.38912	0.69212	1.24077
H33	0.45663	0.50551	3.06349	H69	0.33917	0.59804	1.48219
B34	0.49435	0.51839	3.38436	H70	0.35928	0.56556	1.34731
H35	0.50823	0.54474	3.34397	H71	0.38818	0.66009	0.88352
B36	0.48732	0.50555	3.66513	H72	0.40809	0.62803	0.75088

Supplementary Table 3. Atomistic coordinates for the AB-stacking mode of B-COF optimized by using the Materials Studio Forcite molecular dynamics module (Universal force fields).

B-COF: Space group symmetry *P*3							
a = *b* = 51.2534 Å, *c* = 10.6697 Å, *α* = *β* = 90º, *γ* = 120º							
	X	Y	Z		X	Y	Z
N1	0.81966	0.45204	-0.01863	N73	1.15745	0.10907	1.14736
C2	0.64305	0.33998	-0.07206	C74	0.97830	0.00931	1.12976
C3	0.67326	0.36331	-0.07314	C75	1.00923	0.03072	1.12950
C4	0.61823	0.34697	-0.06161	C76	0.95546	0.01912	1.13307
C5	0.74882	0.37873	0.01863	C77	1.08334	0.04049	1.21183
C6	0.77839	0.40222	0.03282	C78	1.11401	0.06198	1.21573
C7	0.78873	0.42915	-0.03335	C79	1.12572	0.08786	1.14105
C8	0.76887	0.43247	-0.11413	C80	1.10614	0.09209	1.06206
C9	0.73909	0.40896	-0.12777	C81	1.07539	0.07064	1.05842
N10	0.45419	0.81953	-0.02450	N82	0.79242	0.48274	1.22886
C11	0.35720	0.69677	-0.02205	C83	0.69127	0.36308	1.28310
C12	0.36321	0.67287	-0.02190	C84	0.69614	0.33844	1.28404
C13	0.38163	0.74938	-0.10708	C85	0.71566	0.41478	1.18911
C14	0.42994	0.78882	-0.02743	C86	0.76705	0.45285	1.24111
C15	0.43053	0.76799	0.05575	C87	0.76838	0.43279	1.32649
C16	0.40706	0.73801	0.05697	C88	0.74378	0.40364	1.34112
C17	0.34616	0.61770	-0.02402	C89	0.67718	0.28287	1.27232
C18	0.37398	0.59475	-0.10933	C90	0.70324	0.25964	1.17278
C19	0.42133	0.45373	-0.07900	C91	0.75065	0.12200	1.10603
C20	0.40821	0.47484	-0.08931	C92	0.73337	0.13935	1.10821
C21	0.39095	0.47746	0.00805	C93	0.71942	0.14167	1.21868

H22	0.38518	0.46368	0.09111	H94	0.71849	0.12950	1.30260
C23	0.38149	0.49877	0.00170	C95	0.70797	0.16135	1.22547
C24	0.38916	0.51812	-0.10180	C96	0.71032	0.17934	1.12235
C25	0.40556	0.51529	-0.20008	C97	0.72253	0.17608	1.01042
C26	0.41487	0.49396	-0.19442	C98	0.73382	0.15635	1.00305
H27	0.42811	0.49316	-0.27166	H99	0.74437	0.15577	0.91621
B28	0.41346	0.43063	0.03902	B100	0.74780	0.09951	1.22026
H29	0.39685	0.42808	0.12183	H101	0.73166	0.09430	1.30806
B30	0.40081	0.41765	-0.10291	B102	0.73405	0.08594	1.08016
H31	0.37471	0.40631	-0.12750	H103	0.70760	0.07147	1.06257
B32	0.42694	0.43939	-0.20527	B104	0.75687	0.11008	0.97402
H33	0.42018	0.44380	-0.30654	H105	0.74734	0.11302	0.87658
B34	0.45572	0.46612	-0.12642	B106	0.78461	0.13890	1.04874
H35	0.47066	0.49082	-0.16815	H107	0.79583	0.16383	1.00793
B36	0.44727	0.46082	0.02447	B108	0.77891	0.13243	1.20095
H37	0.45571	0.48154	0.09475	H109	0.78580	0.15251	1.27341
C38	0.52250	0.35541	-0.07816	C110	0.86665	0.03792	1.08013
C39	0.38169	0.54237	-0.10834	C111	0.70326	0.20380	1.13166
C40	0.44990	0.41341	-0.06154	C112	0.78784	0.08990	1.10151
C41	0.46539	0.39440	-0.05273	C113	0.80731	0.07482	1.10057
C42	0.48866	0.40185	0.03541	C114	0.83193	0.08458	1.18379
H43	0.49424	0.41928	0.10458	H115	0.83692	0.10204	1.25214
C44	0.50641	0.38829	0.03135	C116	0.85105	0.07251	1.17879
C45	0.50125	0.36655	-0.05992	C117	0.84610	0.05016	1.09091
C46	0.47678	0.35714	-0.14138	C118	0.82141	0.03970	1.00991
C47	0.45909	0.37091	-0.13832	C119	0.80228	0.05185	1.01426

H48	0.44132	0.36372	-0.20668	H120	0.78392	0.04332	0.94822
B49	0.45763	0.43617	-0.17935	B121	0.79058	0.11204	0.98720
H50	0.47435	0.43776	-0.26035	H122	0.80686	0.11631	0.90048
B51	0.47020	0.44954	-0.03750	B123	0.80417	0.12598	1.12734
H52	0.49635	0.46136	-0.01349	H124	0.83060	0.14086	1.14447
B53	0.44412	0.42756	0.06481	B125	0.78143	0.10160	1.23341
H54	0.45057	0.42278	0.16607	H126	0.79068	0.09811	1.33061
B55	0.41549	0.40074	-0.01419	B127	0.75384	0.07276	1.15839
H56	0.40091	0.37600	0.02767	H128	0.74285	0.04783	1.19907
B57	0.42365	0.40623	-0.16500	B129	0.75927	0.07933	1.00650
H58	0.41436	0.38584	-0.23701	H130	0.75142	0.05942	0.93276
H59	0.41170	0.53005	-0.28078	H131	0.72448	0.18979	0.92974
H60	0.36882	0.50057	0.07914	H132	0.69862	0.16352	1.31328
H61	0.52489	0.39566	0.09703	H133	0.86990	0.08095	1.24307
H62	0.47221	0.34008	-0.21150	H134	0.81717	0.02238	0.94156
H63	0.60058	0.39399	0.09562	H135	0.94156	0.07033	1.27722
H64	0.58806	0.31231	-0.19095	H136	0.92575	-0.01341	0.99597
H65	0.54693	0.32326	-0.16666	H137	0.88814	0.00269	1.00309
H66	0.64161	0.38314	0.07259	H138	0.97913	0.05408	1.27178
H67	0.67835	0.38651	-0.07192	H139	1.01638	0.05450	1.13022
H68	0.38634	0.67768	-0.02235	H140	0.71896	0.34238	1.28237
H69	0.31438	0.59199	0.12263	H141	0.64299	0.25451	1.40691
H70	0.32576	0.55074	0.11982	H142	0.65104	0.21136	1.38082
H71	0.37960	0.63705	-0.17125	H143	0.71270	0.30440	1.13431
H72	0.39079	0.59597	-0.17424	H144	0.72023	0.26151	1.10730

Supplementary Table 4. Atomistic coordinates for the ABC-stacking mode of B-COF optimized by using the Materials Studio Forcite molecular dynamics module (Universal force fields).

B-COF: Space group symmetry *P*1							
a = *b* = 51.2534 Å, *c* = 16.0047 Å, *α* = *β* = 90º, *γ* = 120º							
	X	Y	Z		X	Y	Z
N1	0.49438	0.07737	0.20775	H325	0.79011	0.30731	1.05777
C2	0.31920	0.01974	0.17132	C326	0.92595	0.48826	0.85413
C3	0.35075	0.03421	0.17252	C327	0.73603	0.16412	1.03533
C4	0.30332	0.03724	0.17737	C328	0.85190	0.35873	0.91242
C5	0.41281	0.02105	0.23145	C329	0.87123	0.39269	0.89598
C6	0.44414	0.03579	0.23944	C330	0.86379	0.41313	0.93313
C7	0.46235	0.06267	0.19640	H331	0.84456	0.40546	0.97408
C8	0.44873	0.07462	0.14442	C332	0.88131	0.44407	0.91942
C9	0.41738	0.05990	0.13642	C333	0.90707	0.45566	0.86890
N10	0.08291	0.48765	0.43532	C334	0.91511	0.43582	0.83288
C11	0.01185	0.36517	0.54264	C335	0.89749	0.40475	0.84613
C12	0.03273	0.35562	0.55431	H336	0.90492	0.39046	0.81794
C13	0.03112	0.40165	0.42461	B337	0.82582	0.33676	0.84723
C14	0.06467	0.45667	0.45937	H338	0.82121	0.34631	0.78454
C15	0.06509	0.44895	0.54298	B339	0.81671	0.34439	0.93950
C16	0.04531	0.42010	0.57284	H340	0.80463	0.35901	0.94490
C17	0.04557	0.31452	0.55426	B341	0.84215	0.34716	1.00794
C18	0.05762	0.28956	0.43545	H342	0.84970	0.36441	1.06483
C19	0.13232	0.17595	0.29464	B343	0.86681	0.34099	0.95795
C20	0.11637	0.19336	0.31930	H344	0.89270	0.35370	0.97748
C21	0.10965	0.19541	0.40389	B345	0.85652	0.33424	0.85871

H22	0.11508	0.18437	0.45281	H346	0.87430	0.34098	0.80361
C23	0.09668	0.21262	0.42745	H347	0.72562	0.19592	0.92068
C24	0.09017	0.22839	0.36718	H348	0.79318	0.20265	1.09324
C25	0.09647	0.22644	0.28324	H349	0.87477	0.45892	0.94909
C26	0.10950	0.20928	0.25944	H350	0.93524	0.44438	0.79501
H27	0.11472	0.20929	0.19415	H351	0.90775	0.54681	0.95549
B28	0.12943	0.14871	0.35370	H352	0.99732	0.59464	0.78278
H29	0.11437	0.14072	0.41518	H353	0.97011	0.54026	0.79369
B30	0.11365	0.14155	0.26030	H354	0.93524	0.60118	0.94738
H31	0.08698	0.12886	0.25237	H355	0.94915	0.64218	0.85791
B32	0.13603	0.16929	0.19693	H356	0.65826	0.02196	1.34117
H33	0.12609	0.17649	0.14005	H357	0.71937	0.07788	1.28530
B34	0.16543	0.19397	0.25157	H358	0.75193	0.12528	1.22179
H35	0.17722	0.22025	0.23727	H359	0.65223	0.06151	1.09745
B36	0.16143	0.18130	0.34851	H360	0.68822	0.10990	1.03105
H37	0.17033	0.19817	0.40632	N361	0.91916	0.50720	0.88718
C38	0.23018	0.07782	0.17400	C362	0.03706	0.68350	0.86090
C39	0.07788	0.24790	0.39010	C363	0.02011	0.65194	0.85883
C40	0.16592	0.14230	0.24863	C364	0.07053	0.69914	0.86591
C41	0.18296	0.12561	0.22796	C365	0.94289	0.58821	0.91228
C42	0.21017	0.13308	0.26936	C366	0.92666	0.55687	0.91882
H43	0.21946	0.15050	0.31651	C367	0.93624	0.53923	0.87676
C44	0.22577	0.11790	0.25169	C368	0.96230	0.55341	0.82688
C45	0.21453	0.09462	0.19253	C369	0.97854	0.58482	0.81996
C46	0.18748	0.08671	0.15164	N370	0.73633	0.91809	1.10955
C47	0.17179	0.10187	0.16920	C371	0.67942	0.98715	1.18890

H48	0.15079	0.09453	0.13690	C372	0.65518	0.96874	1.24051
B49	0.16879	0.16926	0.18908	C373	0.68135	0.94890	1.09481
H50	0.18376	0.17646	0.12738	C374	0.72102	0.93455	1.12679
B51	0.18442	0.17686	0.28271	C375	0.72837	0.95160	1.20018
H52	0.21108	0.19026	0.29044	C376	0.71677	0.97050	1.21731
B53	0.16216	0.14884	0.34587	C377	0.60333	0.96238	1.23481
H54	0.17216	0.14089	0.40106	C378	0.57014	0.95862	1.11495
B55	0.13270	0.12418	0.29106	C379	0.38116	0.86839	0.96341
H56	0.12090	0.09789	0.30517	C380	0.41431	0.88700	0.98957
B57	0.13666	0.13693	0.19443	C381	0.42234	0.89121	1.07499
H58	0.12713	0.12057	0.13584	H382	0.40537	0.88225	1.12343
H59	0.09175	0.23865	0.23598	C383	0.45247	0.90685	1.09973
H60	0.09227	0.21400	0.49301	C384	0.47552	0.91897	1.03986
H61	0.24650	0.12427	0.28496	C385	0.46794	0.91597	0.95509
H62	0.17832	0.06857	0.10632	C386	0.43775	0.90042	0.93011
H63	0.30625	0.09574	0.28009	H387	0.43309	0.89901	0.86408
H64	0.26831	0.01025	0.09260	B388	0.35612	0.87296	1.01334
H65	0.24006	0.03621	0.10694	H389	0.36286	0.89056	1.06915
H66	0.33437	0.06951	0.26732	B390	0.36500	0.88442	0.91549
H67	0.36303	0.05726	0.17444	H391	0.37850	0.91053	0.89846
H68	0.05659	0.37182	0.55004	B392	0.37145	0.85989	0.86566
H69	0.03828	0.28829	0.66920	H393	0.38892	0.86715	0.80965
H70	0.05266	0.25397	0.61539	B394	0.36711	0.83367	0.93307
H71	0.05362	0.32920	0.42430	H395	0.38228	0.82225	0.93031
H72	0.06375	0.29003	0.37052	B396	0.35739	0.84148	1.02406
N73	0.25537	0.08442	0.21015	H397	0.36482	0.83510	1.08777

C74	0.31610	0.96931	0.17139	C398	0.18476	0.76938	0.85328
C75	0.30163	0.98637	0.16951	C399	0.50748	0.93382	1.06424
C76	0.29829	0.93583	0.17514	C400	0.31426	0.83130	0.91499
C77	0.31412	0.06233	0.23078	C401	0.28060	0.81360	0.89341
C78	0.29829	0.07779	0.23996	C402	0.26134	0.78485	0.92793
C79	0.27145	0.06859	0.19618	H403	0.26989	0.77369	0.96708
C80	0.26075	0.04378	0.14169	C404	0.23025	0.77019	0.91475
C81	0.27656	0.02827	0.13250	C405	0.21751	0.78373	0.86615
N82	0.84603	0.26355	0.45356	C406	0.23629	0.81195	0.83047
C83	0.96894	0.31251	0.55297	C407	0.26736	0.82676	0.84405
C84	0.97949	0.34338	0.54711	H408	0.28080	0.84898	0.81753
C85	0.93092	0.29084	0.43971	B409	0.33913	0.82651	0.86471
C86	0.87697	0.27513	0.47651	H410	0.33214	0.80885	0.80903
C87	0.88572	0.28507	0.55848	B411	0.33075	0.81525	0.96252
C88	0.91480	0.29444	0.58689	H412	0.31844	0.78924	0.98006
C89	0.02147	0.39682	0.51644	B413	0.32390	0.83954	1.01209
C90	0.04662	0.43434	0.40126	H414	0.30557	0.83136	1.06598
C91	0.15346	0.62516	0.27449	B415	0.32857	0.86611	0.94509
C92	0.13583	0.59153	0.29651	H416	0.31365	0.87792	0.94917
C93	0.14097	0.58046	0.37188	B417	0.33806	0.85806	0.85385
H94	0.15809	0.59501	0.41550	H418	0.33073	0.86401	0.78931
C95	0.12364	0.55007	0.39339	H419	0.48544	0.92548	0.90799
C96	0.10066	0.52987	0.34034	H420	0.45767	0.90903	1.16582
C97	0.09567	0.54028	0.26470	H421	0.21617	0.74843	0.94363
C98	0.11295	0.57067	0.24297	H422	0.22682	0.82287	0.79333
H99	0.10798	0.57781	0.18445	H423	0.12615	0.69384	0.96077

B100	0.18000	0.64955	0.33563	H424	0.07875	0.73467	0.78283
H101	0.18771	0.64325	0.39881	H425	0.13313	0.76130	0.79190
B102	0.18824	0.64107	0.24330	H426	0.07166	0.66736	0.95330
H103	0.20108	0.62718	0.23740	H427	0.03083	0.63962	0.85919
B104	0.16114	0.63566	0.17731	H428	0.65017	0.94601	1.25409
H105	0.15408	0.61793	0.12181	H429	0.58244	0.96409	1.35346
B106	0.13588	0.64036	0.22920	H430	0.53410	0.95027	1.29655
H107	0.10975	0.62615	0.21285	H431	0.61556	0.96725	1.10312
B108	0.14744	0.64870	0.32715	H432	0.56625	0.95629	1.04818
H109	0.12996	0.64047	0.38359	N433	0.83057	0.41007	1.55325
C110	0.25662	0.82195	0.16656	C434	0.65477	0.34913	1.53024
C111	0.08145	0.49796	0.36256	C435	0.68640	0.36404	1.52959
C112	0.18749	0.69252	0.22944	C436	0.63868	0.36637	1.53811
C113	0.20482	0.72630	0.20928	C437	0.74977	0.35251	1.58199
C114	0.19435	0.74558	0.23724	C438	0.78115	0.36791	1.58792
H115	0.17378	0.73718	0.27265	C439	0.79849	0.39465	1.54347
C116	0.21075	0.77652	0.22226	C440	0.78398	0.40581	1.49208
C117	0.23833	0.78923	0.17982	C441	0.75256	0.39042	1.48614
C118	0.24911	0.77053	0.15193	N442	0.41557	0.82020	1.78256
C119	0.23266	0.73948	0.16645	C443	0.34441	0.69965	1.89331
H120	0.24231	0.72608	0.14574	C444	0.36626	0.69111	1.88325
B121	0.16119	0.66848	0.16812	C445	0.35120	0.73773	1.78811
H122	0.15464	0.67637	0.10544	C446	0.39532	0.79017	1.81112
B123	0.15252	0.67629	0.26078	C447	0.40098	0.78143	1.88844
H124	0.13856	0.68901	0.26679	C448	0.38092	0.75383	1.92451
B125	0.17985	0.68209	0.32636	C449	0.38081	0.65080	1.85751

H126	0.18718	0.70006	0.38135	C450	0.38965	0.61890	1.75358
B127	0.20513	0.67739	0.27446	C451	0.46820	0.50605	1.65171
H128	0.23124	0.69186	0.29089	C452	0.45199	0.52356	1.67246
B129	0.19348	0.66876	0.17673	C453	0.45148	0.53297	1.75519
H130	0.21057	0.67616	0.11960	H454	0.46174	0.52733	1.80583
H131	0.07806	0.52492	0.22278	C455	0.43793	0.55007	1.77405
H132	0.12811	0.54247	0.45198	C456	0.42436	0.55808	1.71077
H133	0.20199	0.79052	0.24514	C457	0.42387	0.54820	1.62916
H134	0.27064	0.78001	0.12041	C458	0.43732	0.53104	1.61014
H135	0.23659	0.87965	0.26549	H459	0.43614	0.52394	1.54619
H136	0.32702	0.92808	0.09637	B460	0.46469	0.47947	1.71431
H137	0.30013	0.87388	0.10683	H461	0.44962	0.47308	1.77576
H138	0.26394	0.93412	0.25805	B462	0.44912	0.47129	1.62055
H139	0.27860	0.97575	0.16847	H463	0.42249	0.45893	1.61208
H140	0.96367	0.35128	0.53734	B464	0.47200	0.49809	1.55530
H141	0.04522	0.41570	0.63534	H465	0.46296	0.50519	1.49620
H142	0.07946	0.46509	0.58409	B466	0.50142	0.52308	1.60902
H143	0.01115	0.38855	0.38400	H467	0.51365	0.54919	1.59221
H144	0.04634	0.44002	0.33626	B468	0.49712	0.51156	1.70718
N145	0.24871	0.84032	0.19768	H469	0.50665	0.52920	1.76314
C146	0.36570	0.01760	0.17351	C470	0.56485	0.40595	1.54112
C147	0.34765	0.98426	0.17250	C471	0.41110	0.57720	1.72717
C148	0.39905	0.03299	0.18012	C472	0.50082	0.47082	1.61312
C149	0.27182	0.92125	0.22339	C473	0.51736	0.45343	1.59528
C150	0.25566	0.88986	0.22947	C474	0.54462	0.46106	1.63664
C151	0.26561	0.87242	0.18803	H475	0.55402	0.47874	1.68302

C152	0.29194	0.88681	0.13937	C476	0.56028	0.44590	1.61923
C153	0.30805	0.91819	0.13276	C477	0.54898	0.42245	1.56057
N154	0.07138	0.25060	0.46670	C478	0.52172	0.41417	1.52048
C155	0.02298	0.32409	0.58270	C479	0.50600	0.42930	1.53775
C156	0.99046	0.30063	0.55797	H480	0.48492	0.42174	1.50573
C157	0.04324	0.30775	0.45999	B481	0.50436	0.49728	1.55027
C158	0.06012	0.27000	0.49209	H482	0.51943	0.50345	1.48889
C159	0.05188	0.26922	0.57556	B483	0.51983	0.50576	1.64396
C160	0.04322	0.28910	0.60689	H484	0.54647	0.51880	1.65209
C161	0.93664	0.29022	0.53351	B485	0.49710	0.47873	1.70911
C162	0.89807	0.27615	0.41877	H486	0.50677	0.47119	1.76609
C163	0.70984	0.19943	0.28386	B487	0.46751	0.45377	1.65537
C164	0.74321	0.21680	0.30851	H488	0.45524	0.42765	1.67209
C165	0.75186	0.21987	0.39341	B489	0.47188	0.46537	1.55733
H166	0.73522	0.21085	0.44268	H490	0.46213	0.44834	1.50020
C167	0.78219	0.23429	0.41639	H491	0.41319	0.55397	1.57971
C168	0.80474	0.24596	0.35514	H492	0.43839	0.55729	1.83791
C169	0.79660	0.24395	0.27105	H493	0.58118	0.45252	1.65189
C170	0.76628	0.22985	0.24795	H494	0.51251	0.39587	1.47566
H171	0.76125	0.22917	0.18240	H495	0.64118	0.42384	1.64537
B172	0.68548	0.20337	0.33944	H496	0.60387	0.34001	1.45211
H173	0.69291	0.21973	0.39838	H497	0.57539	0.36559	1.46937
B174	0.69387	0.21696	0.24339	H498	0.66952	0.39791	1.62969
H175	0.70770	0.24339	0.23079	H499	0.69845	0.38709	1.53229
B176	0.69940	0.19328	0.18605	H500	0.38962	0.70799	1.87277
H177	0.71633	0.20163	0.12871	H501	0.38248	0.63569	1.98507

B178	0.69501	0.16542	0.24705	H502	0.39866	0.60087	1.94847
H179	0.70968	0.15360	0.23841	H503	0.38006	0.65403	1.72226
B180	0.68618	0.17142	0.34162	H504	0.39227	0.61441	1.68899
H181	0.69390	0.16354	0.40211	N505	0.59029	0.41283	1.57607
C182	0.51326	0.10271	0.17277	C506	0.65333	0.30078	1.52558
C183	0.83675	0.25919	0.37675	C507	0.63846	0.31753	1.52752
C184	0.64258	0.16435	0.23820	C508	0.63583	0.26736	1.52859
C185	0.60881	0.14725	0.21776	C509	0.64930	0.39097	1.59323
C186	0.58937	0.11856	0.25245	C510	0.63334	0.40623	1.60409
H187	0.59772	0.10754	0.29286	C511	0.60653	0.39726	1.56045
C188	0.55839	0.10372	0.23735	C512	0.59601	0.37295	1.50421
C189	0.54591	0.11715	0.18730	C513	0.61196	0.35767	1.49324
C190	0.56483	0.14542	0.15209	N514	0.18056	0.59585	1.80327
C191	0.59580	0.16041	0.16749	C515	0.30382	0.64566	1.89550
H192	0.60930	0.18263	0.14114	C516	0.31238	0.67620	1.89939
B193	0.66677	0.16028	0.18226	C517	0.26538	0.62387	1.78496
H194	0.65909	0.14390	0.12337	C518	0.21149	0.60687	1.82532
B195	0.65888	0.14686	0.27830	C519	0.22035	0.61504	1.90827
H196	0.64617	0.12048	0.29095	C520	0.24947	0.62378	1.93527
B197	0.65291	0.17032	0.33536	C521	0.35194	0.73174	1.88171
H198	0.63495	0.16127	0.39022	C522	0.37006	0.77018	1.76418
B199	0.65758	0.19847	0.27468	C523	0.48830	0.95668	1.61833
H200	0.64305	0.21049	0.28456	C524	0.47012	0.92306	1.63976
B201	0.66629	0.19230	0.18000	C525	0.47750	0.91094	1.70909
H202	0.65899	0.20005	0.11852	H526	0.49720	0.92444	1.74664
H203	0.81377	0.25312	0.22296	C527	0.45908	0.88091	1.73257

H204	0.78794	0.23576	0.48199	C528	0.43295	0.86213	1.68700
H205	0.54421	0.08188	0.26565	C529	0.42613	0.87335	1.61591
H206	0.55552	0.15620	0.11368	C530	0.44448	0.90336	1.59227
H207	0.45374	0.02692	0.27781	H531	0.43812	0.91117	1.53736
H208	0.40800	0.06892	0.09780	B532	0.51569	0.98040	1.67847
H209	0.46226	0.09534	0.11021	H533	0.52335	0.97356	1.74083
H210	0.39932	0.00060	0.26685	B534	0.52275	0.97173	1.58483
H211	0.35853	0.97209	0.17408	H535	0.53483	0.95719	1.57679
H212	0.98170	0.28139	0.60222	B536	0.49536	0.96726	1.52120
H213	0.92018	0.30119	0.64779	H537	0.48719	0.94946	1.46557
H214	0.87039	0.28532	0.59909	B538	0.47119	0.97288	1.57575
H215	0.94142	0.27970	0.40489	H539	0.44490	0.95961	1.56115
H216	0.89138	0.26839	0.35530	B540	0.48361	0.98067	1.67307
N217	0.16611	0.74394	0.88819	H541	0.46662	0.97275	1.73062
C218	0.98861	0.68459	0.85941	C542	0.59314	0.15339	1.51150
C219	0.02201	0.70005	0.86045	C543	0.41199	0.83108	1.71324
C220	0.97269	0.70212	0.86545	C544	0.52363	0.02406	1.57437
C221	0.08480	0.68761	0.91682	C545	0.54133	0.05787	1.55508
C222	0.11619	0.70247	0.92287	C546	0.53326	0.07773	1.59288
C223	0.13399	0.72914	0.87857	H547	0.51455	0.06970	1.63578
C224	0.11991	0.74076	0.82715	C548	0.54968	0.10862	1.57794
C225	0.08847	0.72589	0.82102	C549	0.57495	0.12074	1.52554
N226	0.74228	0.15153	1.09634	C550	0.58348	0.10148	1.48809
C227	0.67141	0.03079	1.21243	C551	0.56695	0.07044	1.50266
C228	0.68627	0.01802	1.17073	H552	0.57482	0.05659	1.47391
C229	0.67646	0.06958	1.10892	B553	0.49650	0.00067	1.51404

C230	0.72269	0.12128	1.12464	H554	0.48981	0.00904	1.45253
C231	0.73143	0.11137	1.19480	B555	0.48904	0.00872	1.60798
C232	0.71248	0.08357	1.23164	H556	0.47602	0.02234	1.61590
C233	0.69920	0.97627	1.15170	B557	0.51659	0.01347	1.67133
C234	0.69928	0.93398	1.07205	H558	0.52484	0.03131	1.72685
C235	0.79969	0.84024	0.97313	B559	0.54085	0.00802	1.61660
C236	0.78177	0.85620	0.99300	H560	0.56713	0.02185	1.63129
C237	0.78619	0.87231	1.06827	B561	0.52828	-0.00018	1.51954
H238	0.80201	0.87367	1.11487	H562	0.54478	0.00666	1.46119
C239	0.77002	0.88702	1.08539	H563	0.40618	0.85901	1.57948
C240	0.74878	0.88591	1.02811	H564	0.46504	0.87257	1.78727
C241	0.74361	0.86946	0.95426	H565	0.54276	0.12303	1.60851
C242	0.75974	0.85472	0.93695	H566	0.60326	0.11050	1.44860
H243	0.75471	0.84205	0.87941	H567	0.57600	0.21087	1.62437
B244	0.79647	0.81325	1.03344	H568	0.66298	0.26007	1.44235
H245	0.78080	0.80582	1.09382	H569	0.63599	0.20570	1.45029
B246	0.78189	0.80586	0.93843	H570	0.60334	0.26535	1.61912
H247	0.75533	0.79280	0.92799	H571	0.61539	0.30651	1.52866
B248	0.80483	0.83394	0.87640	H572	0.29536	0.68286	1.89917
H249	0.79575	0.84185	0.81890	H573	0.38528	0.74907	1.98332
B250	0.83334	0.85869	0.93330	H574	0.41981	0.79618	1.92131
H251	0.84508	0.88498	0.91932	H575	0.32752	0.72892	1.76787
B252	0.82842	0.84584	1.03016	H576	0.36546	0.77700	1.70456
H253	0.83751	0.86255	1.08811	N577	0.58654	0.17187	1.54824
C254	0.89930	0.74256	0.86423	C578	0.70170	0.34770	1.52727
C255	0.73198	0.90187	1.04318	C579	0.68496	0.31607	1.52479

C256	0.83448	0.80723	0.93266	C580	0.73511	0.36363	1.53124
C257	0.85179	0.79056	0.91412	C581	0.61042	0.25260	1.58069
C258	0.87989	0.79945	0.95235	C582	0.59431	0.22123	1.58558
H259	0.88977	0.81796	0.99610	C583	0.60329	0.20396	1.53916
C260	0.89563	0.78425	0.93595	C584	0.62860	0.21853	1.48667
C261	0.88363	0.75951	0.88150	C585	0.64470	0.24996	1.48134
C262	0.85570	0.75018	0.84402	N586	0.40980	0.58649	1.80155
C263	0.83989	0.76532	0.86033	C587	0.35749	0.65902	1.88064
H264	0.81814	0.75675	0.83117	C588	0.32717	0.63696	1.88935
B265	0.83781	0.83426	0.87232	C589	0.37390	0.63633	1.77054
H266	0.85352	0.84169	0.81204	C590	0.39762	0.60581	1.81851
B267	0.85230	0.84177	0.96720	C591	0.39386	0.61149	1.90174
H268	0.87883	0.85550	0.97740	C592	0.38418	0.63164	1.92315
B269	0.82950	0.81359	1.02914	C593	0.27184	0.62207	1.87760
H270	0.83908	0.80572	1.08562	C594	0.23250	0.60909	1.76544
B271	0.80080	0.78886	0.97241	C595	0.04524	0.53461	1.62892
H272	0.78901	0.76256	0.98646	C596	0.07843	0.55209	1.65500
B273	0.80584	0.80173	0.87543	C597	0.08661	0.55476	1.74029
H274	0.79726	0.78547	0.81645	H598	0.06969	0.54574	1.78887
H275	0.72709	0.86811	0.90957	C599	0.11678	0.56852	1.76449
H276	0.77421	0.89932	1.14350	C600	0.13960	0.58011	1.70410
H277	0.91710	0.79177	0.96644	C601	0.13196	0.57875	1.61980
H278	0.84593	0.73092	0.80243	C602	0.10181	0.56515	1.59542
H279	0.97661	0.76162	0.96514	H603	0.09715	0.56471	1.52966
H280	0.93688	0.67449	0.78344	B604	0.02034	0.53751	1.68491
H281	0.90876	0.70050	0.79826	H605	0.02724	0.55337	1.74486

H282	1.00459	0.73522	0.95215	B606	0.02885	0.55194	1.58977
H283	0.03412	0.72310	0.86211	H607	0.04224	0.57848	1.57888
H284	0.70405	0.03145	1.12669	B608	0.03517	0.52916	1.53074
H285	0.72345	0.98329	1.27063	H609	0.05234	0.53818	1.47378
H286	0.74306	0.95064	1.24184	B610	0.03114	0.50099	1.58973
H287	0.67442	0.95598	1.03735	H611	0.04632	0.48983	1.57926
H288	0.69309	0.92045	1.01581	B612	0.02177	0.50591	1.68486
N289	0.92502	0.74977	0.89819	H613	0.02961	0.49782	1.74468
C290	0.98674	0.63603	0.85810	C614	0.84889	0.43531	1.51692
C291	0.97220	0.65303	0.85767	C615	0.17143	0.59261	1.72643
C292	0.96907	0.60260	0.86287	C616	0.97819	0.49882	1.58147
C293	0.98399	0.72771	0.91705	C617	0.94450	0.48123	1.56069
C294	0.96823	0.74329	0.92634	C618	0.92534	0.45287	1.59753
C295	0.94095	0.73371	0.88448	H619	0.93394	0.44249	1.63958
C296	0.92975	0.70840	0.83169	C620	0.89437	0.43762	1.58278
C297	0.94550	0.69282	0.82227	C621	0.88159	0.45027	1.53088
N298	0.51554	0.93777	1.14210	C622	0.90021	0.47822	1.49359
C299	0.63463	0.97914	1.27506	C623	0.93121	0.49363	1.50859
C300	0.64822	0.01356	1.27860	H624	0.94447	0.51559	1.48055
C301	0.60213	0.97301	1.14588	B625	1.00291	0.49583	1.52495
C302	0.54607	0.95067	1.17065	H626	0.99569	0.47996	1.46504
C303	0.55138	0.95428	1.25673	B627	0.99494	0.48157	1.62010
C304	0.57955	0.96235	1.28995	H628	0.98257	0.45509	1.63091
C305	0.68089	0.06339	1.20012	B629	0.98820	0.50417	1.67892
C306	0.69513	0.10221	1.08518	H630	0.97009	0.49445	1.73351
C307	0.81317	0.29098	0.95000	B631	0.99246	0.53258	1.62005

C308	0.79393	0.25714	0.96853	H632	0.97740	0.54399	1.63073
C309	0.80363	0.24291	1.02631	B633	1.00171	0.52748	1.52493
H310	0.82538	0.25509	1.05623	H634	0.99442	0.53552	1.46402
C311	0.78525	0.21258	1.04726	H635	0.14941	0.58790	1.57252
C312	0.75676	0.19551	1.01053	H636	0.12217	0.56950	1.83025
C313	0.74742	0.20887	0.95041	H637	0.88042	0.41609	1.61297
C314	0.76579	0.23917	0.92910	H638	0.89065	0.48844	1.45391
H315	0.75760	0.24832	0.88184	H639	0.79144	0.35967	1.62593
B316	0.83945	0.31325	1.01507	H640	0.74246	0.39889	1.44821
H317	0.84458	0.30476	1.07883	H641	0.79685	0.42645	1.45696
B318	0.84823	0.30512	0.92291	H642	0.73693	0.33223	1.61859
H319	0.85955	0.28980	0.91718	H643	0.69597	0.30401	1.52399
B320	0.82283	0.30247	0.85439	H644	0.32044	0.61334	1.88381
H321	0.81524	0.28515	0.79768	H645	0.25478	0.62885	1.99711
B322	0.79836	0.30894	0.90411	H646	0.20501	0.61425	1.95078
H323	0.77259	0.29733	0.88436	H647	0.27606	0.61369	1.74664
B324	0.80842	0.31519	1.00353	H648	0.22573	0.60259	1.70125

Supplementary Table 5. Experimental (Exp.) and calculated (Cal.) elemental analysis of B-COF.

Sample	C (%)	N (%)	H (%)	B[b] (%)
Cal[a].	70.05	5.11	5.14	19.70
Exp.	69.70	4.70	5.05	20.55

[a] Calculated with the unit of B-COF structure. [b] B (%) = 100% - C (%) - N (%) - H (%).

Supplementary Table 6. Particle deposition energy spectrum of 1-µm B-COF sphere.

Particles	Max (keV)	Min (keV)	Average (keV)	Counts
^{7}Li	9.38E+00	5.73E-34	2.59E+00	821
Alpha	5.55E+00	2.13E-40	1.31E+00	821
Proton	4.41E-01	3.71E-20	6.72E-03	66
^{12}C	7.28E-05	1.26E-06	4.65E-05	19
^{11}B	6.22E-05	7.34E-06	3.96E-05	6
^{14}N	5.48E-05	1.19E-05	3.15E-05	3
^{14}C	4.26E+00	4.22E+00	4.24E+00	2

Supplementary Table 7. Percentage of energy deposited and remaining by particles in 1-μm B-COF sphere.

Particles	Deposited energy (%)	Remaining energy (%)
^{7}Li	0.31	99.69
Alpha	0.09	99.91

Supplementary Table 8. Particle deposition energy spectrum of 64 1-μm B-COF spheres.

Particles	Max (keV)	Min (keV)	Average (keV)	Counts
^{7}Li	1.13E+01	1.22E-67	3.52E+00	12439
Alpha	6.44E+00	2.35E-134	1.88E+00	12241
Proton	9.23E-01	3.85E-07	8.84E-03	511
^{12}C	1.60E-04	3.34E-06	4.02E-05	142
^{11}B	1.02E-04	2.15E-06	4.23E-05	42
Deuteron	1.32E+00	3.78E-36	1.41E-01	22
^{14}N	1.28E-04	4.11E-06	3.14E-05	20
^{14}C	4.36E+00	1.63E-01	2.22E+00	8
^{10}B	8.20E-05	2.96E-06	3.78E-05	5
Electron	9.68E-01	2.87E-02	3.81E-01	4
^{13}C	3.13E-05	3.21E-06	1.73E-05	2
Gamma	2.65E-02	2.65E-02	2.65E-02	2

Supplementary Table 9. Percentage of energy deposited and remaining by particles in 64 1-μm B-COF spheres.

Particles	Deposited energy (%)	Remaining energy (%)
^{7}Li	0.42	99.58
Alpha	0.13	99.87

Supplementary Table 10. *P* values in figure 4D, F (two-tailed unpaired Student's *t*-test).

Tumors	Group	B16F10 Boron capsule+N		MC38 Boron capsule+N	
		P value	Summary	*P* value	Summary
primary	PEG-B-COF+N	0.0581	ns	0.8184	ns
	Imiquimod+N	< 0.0001	****	< 0.0001	****
	PBS+N	< 0.0001	****	< 0.0001	****
	Boron capsule	< 0.0001	****	< 0.0001	****
	PEG-B-COF	< 0.0001	****	< 0.0001	****
	Imiquimod	< 0.0001	****	< 0.0001	****
	PBS	< 0.0001	****	0.8184	ns

Supplementary Table 11. P values in figure 5G-K (one-way analysis of variance (ANOVA) followed by Tukey's HSD post hoc test).

Group	CD4+		CD8+		Foxp3+		M1		M1/M2 ratio	
	P Value	Summary	*P* Value	Summary	*P* Value	Summary	*P* Value	Summary	*P* Value	Summary
PBS vs. PBS+N	0.0003	***	<0.0001	****	0.9397	ns	0.0100	**	0.8401	ns
PBS vs. Imiquimod+N	<0.0001	****	<0.0001	****	0.871	ns	<0.0001	****	0.0009	***
PBS vs. B-COF+N	<0.0001	****	<0.0001	****	<0.0001	****	0.0031	**	0.8177	ns
PBS vs. Boron capsule+N	<0.0001	****	<0.0001	****	<0.0001	****	<0.0001	****	<0.0001	****
Imiquimod vs. PBS+N	0.1491	ns	<0.0001	****	0.9991	ns	0.3502	ns	0.9996	ns
Imiquimod vs. Imiquimod+N	0.0057	**	<0.0001	****	0.5432	ns	0.0002	***	0.0774	ns
Imiquimod vs. B-COF+N	<0.0001	****	<0.0001	****	<0.0001	****	0.1537	ns	0.9998	ns
Imiquimod vs. Boron capsule+N	<0.0001	****	<0.0001	****	<0.0001	****	<0.0001	****	<0.0001	****
PEG-B-COF vs. PBS+N	0.007	**	<0.0001	****	0.9944	ns	0.0127	*	0.8586	ns
PEG-B-COF vs. Imiquimod+N	0.0002	***	<0.0001	****	0.0586	ns	<0.0001	****	0.0010	***
PEG-B-COF vs. B-COF+N	<0.0001	****	<0.0001	****	<0.0001	****	0.0040	**	0.8374	ns
PEG-B-COF vs. Boron capsule+N	<0.0001	****	<0.0001	****	<0.0001	****	<0.0001	****	<0.0001	****
Boron capsule vs. PBS+N	0.2835	ns	<0.0001	****	>0.9999	ns	0.1551	ns	0.9999	ns
Boron capsule vs. Imiquimod+N	0.0137	*	<0.0001	****	0.4259	ns	<0.0001	****	0.0656	ns
Boron capsule vs. B-COF+N	<0.0001	****	<0.0001	****	<0.0001	****	0.0581	ns	>0.9999	ns
Boron capsule vs. Boron capsule+N	<0.0001	****	<0.0001	****	<0.0001	****	<0.0001	****	<0.0001	****
PBS+N vs. Imiquimod+N	0.8087	ns	0.1553	ns	0.2453	ns	0.0524	ns	0.0267	*
PBS+N vs. B-COF+N	<0.0001	****	<0.0001	****	<0.0001	****	0.9996	ns	>0.9999	ns
PBS+N vs. Boron capsule+N	<0.0001	****	<0.0001	****	<0.0001	****	0.0025	**	<0.0001	****
Imiquimod+N vs. B-COF+N	<0.0001	****	<0.0001	****	<0.0001	****	0.1414	ns	0.0297	*
Imiquimod+N vs. Boron capsule+N	<0.0001	****	<0.0001	****	<0.0001	****	0.8851	ns	<0.0001	****
B-COF+N vs. Boron capsule+N	<0.0001	****	0.0017	**	>0.9999	ns	0.0080	**	<0.0001	****

Supplementary Table 12. *P* values in Supplementary Figure 25 (One-way analysis of variance (ANOVA) followed by Tukey's HSD post hoc test).

Group	CD3+		M2	
	P Value	Summary	*P* Value	Summary
PBS vs. Imiquimod	0.988	ns	0.0032	**
PBS vs. PEG-B-COF	0.5999	ns	>0.9999	ns
PBS vs. Boron capsule	0.0161	*	0.0015	**
PBS vs. PBS+N	0.009	**	0.9998	ns
PBS vs. Imiquimod+N	<0.0001	****	0.0121	*
PBS vs. B-COF+N	<0.0001	****	>0.9999	ns
PBS vs. Boron capsule+N	<0.0001	****	<0.0001	****
Imiquimod vs. PEG-B-COF	0.9711	ns	0.0016	**
Imiquimod vs. Boron capsule	0.1023	ns	>0.9999	ns
Imiquimod vs. PBS+N	0.0615	ns	0.009	**
Imiquimod vs. Imiquimod+N	<0.0001	****	0.999	ns
Imiquimod vs. B-COF+N	<0.0001	****	0.0047	**
Imiquimod vs. Boron capsule+N	<0.0001	****	0.6368	ns
PEG-B-COF vs. Boron capsule	0.5175	ns	0.0008	***
PEG-B-COF vs. PBS+N	0.374	ns	0.996	ns
PEG-B-COF vs. Imiquimod+N	<0.0001	****	0.0064	**
PEG-B-COF vs. B-COF+N	<0.0001	****	0.9998	ns
PEG-B-COF vs. Boron capsule+N	<0.0001	****	<0.0001	****
Boron capsule vs. PBS+N	>0.9999	ns	0.0044	**
Boron capsule vs. Imiquimod+N	0.0112	*	0.9866	ns
Boron capsule vs. B-COF+N	<0.0001	****	0.0023	**
Boron capsule vs. Boron capsule+N	<0.0001	****	0.8099	ns
PBS+N vs. Imiquimod+N	0.0199	*	0.0329	*
PBS+N vs. B-COF+N	<0.0001	****	>0.9999	ns
PBS+N vs. Boron capsule+N	<0.0001	****	0.0001	***
Imiquimod+N vs. B-COF+N	<0.0001	****	0.0178	*
Imiquimod+N vs. Boron capsule+N	<0.0001	****	0.3112	ns
B-COF+N vs. Boron capsule+N	0.0017	**	<0.0001	****

Supplementary Table 13. *P* values in figure 6C (One-way analysis of variance (ANOVA) followed by Tukey's HSD post hoc test).

Group	TNF-α		IL-12p70		IL-6	
	P Value	Summary	*P* Value	Summary	*P* Value	Summary
PBS vs. Imiquimod	0.0036	**	<0.0001	****	0.0035	**
PBS vs. PEG-B-COF	>0.9999	ns	>0.9999	ns	0.9786	ns
PBS vs. Boron capsule	0.0038	**	<0.0001	****	0.0002	***
PBS vs. PBS+N	0.0471	*	0.0306	*	0.2507	ns
PBS vs. Imiquimod+N	<0.0001	****	<0.0001	****	<0.0001	****
PBS vs. B-COF+N	<0.0001	****	<0.0001	****	<0.0001	****
PBS vs. Boron capsule+N	<0.0001	****	<0.0001	****	<0.0001	****
Imiquimod vs. PEG-B-COF	0.0039	**	<0.0001	****	0.0462	*
Imiquimod vs. Boron capsule	>0.9999	ns	0.9968	ns	0.975	ns
Imiquimod vs. PBS+N	0.9785	ns	0.1176	ns	0.6619	ns
Imiquimod vs. Imiquimod+N	<0.0001	****	0.0018	**	0.002	**
Imiquimod vs. B-COF+N	<0.0001	****	<0.0001	****	<0.0001	****
Imiquimod vs. Boron capsule+N	<0.0001	****	<0.0001	****	<0.0001	****
PEG-B-COF vs. Boron capsule	0.0041	**	<0.0001	****	0.0032	**
PEG-B-COF vs. PBS+N	0.0505	ns	0.0321	*	0.8087	ns
PEG-B-COF vs. Imiquimod+N	<0.0001	****	<0.0001	****	<0.0001	****
PEG-B-COF vs. B-COF+N	<0.0001	****	<0.0001	****	<0.0001	****
PEG-B-COF vs. Boron capsule+N	<0.0001	****	<0.0001	****	<0.0001	****
Boron capsule vs. PBS+N	0.9809	ns	0.0223	*	0.1467	ns
Boron capsule vs. Imiquimod+N	<0.0001	****	0.0132	*	0.0307	*
Boron capsule vs. B-COF+N	<0.0001	****	<0.0001	****	<0.0001	****
Boron capsule vs. Boron capsule+N	<0.0001	****	<0.0001	****	<0.0001	****
PBS+N vs. Imiquimod+N	<0.0001	****	<0.0001	****	<0.0001	****
PBS+N vs. B-COF+N	<0.0001	****	<0.0001	****	<0.0001	****
PBS+N vs. Boron capsule+N	<0.0001	****	<0.0001	****	<0.0001	****
Imiquimod+N vs. B-COF+N	<0.0001	****	<0.0001	****	<0.0001	****
Imiquimod+N vs. Boron capsule+N	<0.0001	****	<0.0001	****	<0.0001	****
B-COF+N vs. Boron capsule+N	<0.0001	****	<0.0001	****	<0.0001	****

Supplementary Table 14. P values in figure 6D-G (One-way analysis of variance (ANOVA) followed by Tukey's HSD post hoc test).

Group	CD3+		CD4+		CD8+		Foxp3+	
	P Value	Summary	*P* Value	Summary	*P* Value	Summary	*P* Value	Summary
PBS vs. PBS+N	>0.9999	ns	0.725	ns	>0.9999	ns	0.9961	ns
PBS vs. Imiquimod+N	0.9995	ns	0.9156	ns	0.9995	ns	0.9996	ns
PBS vs. B-COF+N	<0.0001	****	<0.0001	****	<0.0001	****	0.7972	ns
PBS vs. Boron capsule+N	<0.0001	****	<0.0001	****	<0.0001	****	<0.0001	****
Imiquimod vs. PBS+N	0.9987	ns	0.7947	ns	>0.9999	ns	>0.9999	ns
Imiquimod vs. Imiquimod+N	>0.9999	ns	0.9503	ns	>0.9999	ns	0.9997	ns
Imiquimod vs. B-COF+N	<0.0001	****	<0.0001	****	<0.0001	****	0.2488	ns
Imiquimod vs. Boron capsule+N	<0.0001	****	<0.0001	****	<0.0001	****	<0.0001	****
PEG-B-COF vs. PBS+N	>0.9999	ns	0.869	ns	>0.9999	ns	0.9999	ns
PEG-B-COF vs. Imiquimod+N	0.9999	ns	0.9781	ns	>0.9999	ns	>0.9999	ns
PEG-B-COF vs. B-COF+N	<0.0001	****	<0.0001	****	<0.0001	****	0.6253	ns
PEG-B-COF vs. Boron capsule+N	<0.0001	****	<0.0001	****	<0.0001	****	<0.0001	****
Boron capsule vs. PBS+N	>0.9999	ns	0.9851	ns	>0.9999	ns	0.9997	ns
Boron capsule vs. Imiquimod+N	>0.9999	ns	0.9997	ns	0.9999	ns	>0.9999	ns
Boron capsule vs. B-COF+N	<0.0001	****	<0.0001	****	<0.0001	****	0.6708	ns
Boron capsule vs. Boron capsule+N	<0.0001	****	<0.0001	****	<0.0001	****	<0.0001	****
PBS+N vs. Imiquimod+N	0.9999	ns	0.9999	ns	>0.9999	ns	>0.9999	ns
PBS+N vs. B-COF+N	<0.0001	****	<0.0001	****	<0.0001	****	0.3819	ns
PBS+N vs. Boron capsule+N	<0.0001	****	<0.0001	****	<0.0001	****	<0.0001	****
Imiquimod+N vs. B-COF+N	<0.0001	****	<0.0001	****	<0.0001	****	0.4995	ns
Imiquimod+N vs. Boron capsule+N	<0.0001	****	<0.0001	****	<0.0001	****	<0.0001	****
B-COF+N vs. Boron capsule+N	0.0019	**	<0.0001	****	<0.0001	****	<0.0001	****

Supplementary Table 15. *P* values in figure 6H, J (two-tailed unpaired Student's *t*-test).

Tumors	Group	B16F10 Boron capsule+N		MC38 Boron capsule+N	
		P value	Summary	*P* value	Summary
Distant	PEG-B-COF+N	< 0.0001	****	0.0015	**
	Imiquimod+N	< 0.0001	****	< 0.0001	****
	PBS+N	< 0.0001	****	< 0.0001	****
	Boron capsule	< 0.0001	****	< 0.0001	****
	PEG-B-COF	< 0.0001	****	< 0.0001	****
	Imiquimod	< 0.0001	****	< 0.0001	****
	PBS	< 0.0001	****	< 0.0001	****
