# Supplementary material for: Localized nuclear reaction breaks boron drug capsules loaded with immune adjuvants for cancer immunotherapy
Source: Nat Commun. 2023 Apr 5;14:1884. doi: 10.1038/s41467-023-37253-x (PMC10076324; doi:10.1038/s41467-023-37253-x)
Supplement: Supplementary file 3 — Reporting Summary [file 41467_2023_37253_MOESM3_ESM.pdf]

nature portfolio

Corresponding author(s): Zhen Gu

Last updated by author(s): Feb 16, 2023

Reporting Summary

Nature Portfolio wishes to improve the reproducibility of the work that we publish. This form provides structure for consistency and transparency in reporting. For further information on Nature Portfolio policies, see our Editorial Policies and the Editorial Policy Checklist.

Statistics

For all statistical analyses, confirm that the following items are present in the figure legend, table legend, main text, or Methods section.

n/a	Confirmed	
☐	☒	The exact sample size (n) for each experimental group/condition, given as a discrete number and unit of measurement
☐	☒	A statement on whether measurements were taken from distinct samples or whether the same sample was measured repeatedly
☐	☒	The statistical test(s) used AND whether they are one- or two-sided<br>*Only common tests should be described solely by name; describe more complex techniques in the Methods section.*
☐	☒	A description of all covariates tested
☐	☒	A description of any assumptions or corrections, such as tests of normality and adjustment for multiple comparisons
☐	☒	A full description of the statistical parameters including central tendency (e.g. means) or other basic estimates (e.g. regression coefficient) AND variation (e.g. standard deviation) or associated estimates of uncertainty (e.g. confidence intervals)
☐	☒	For null hypothesis testing, the test statistic (e.g. F, t, r) with confidence intervals, effect sizes, degrees of freedom and P value noted<br>*Give P values as exact values whenever suitable.*
☒	☐	For Bayesian analysis, information on the choice of priors and Markov chain Monte Carlo settings
☒	☐	For hierarchical and complex designs, identification of the appropriate level for tests and full reporting of outcomes
☒	☐	Estimates of effect sizes (e.g. Cohen's d, Pearson's r), indicating how they were calculated

Our web collection on statistics for biologists contains articles on many of the points above.

Software and code

Policy information about availability of computer code

Data collection	Nuclear magnetic resonance (NMR) spectra were recorded on Bruker AVANCE 400 MHz spectrometer. Confocal fluorescence images were recorded on an A1R-si Laser Scanning Confocal Microscope (Nikon, Japan). Transmission electron microscopy (TEM) images were recorded on a JEM-2100F microscope (JEOL Ltd., Japan). The zeta potential and size were determined by Malvern Mastersizer 2000 (Zetasizer Nano ZS90, Malvern Instruments Ltd, UK). PET images were taken on a small animal PET / CT imaging system (Mediso nanoScan® PET122S). The boron concentration was determined by inductively coupled plasma-atomic emission spectroscopy (Prodigy 7, Leeman). H&E staining: 3Dhistech (3DHISTECH)
Data analysis	All statistical analyses were performed on Graphpad Prism (version 8) and Origin (2018) . All the flow cytometry data were processed using FlowJo (version 10.4.0, Becton, Dickinson & Company). MestReNova 7 was used for NMR analysis. Nucline NanoScan software (InterViewTM FUSION, Mediso Medical Imaging Systems) was used to determine the radioactivity concentration. Immunofluorescence and H&E staining: CaseViewer v2.4

For manuscripts utilizing custom algorithms or software that are central to the research but not yet described in published literature, software must be made available to editors and reviewers. We strongly encourage code deposition in a community repository (e.g. GitHub). See the Nature Portfolio guidelines for submitting code & software for further information.

Data

Policy information about availability of data

All manuscripts must include a data availability statement. This statement should provide the following information, where applicable:

- Accession codes, unique identifiers, or web links for publicly available datasets
- A description of any restrictions on data availability
- For clinical datasets or third party data, please ensure that the statement adheres to our policy

All data that support the plots within this paper and other findings of this study are available from the corresponding authors upon reasonable request.

Field-specific reporting

Please select the one below that is the best fit for your research. If you are not sure, read the appropriate sections before making your selection.

☒ Life sciences ☐ Behavioural & social sciences ☐ Ecological, evolutionary & environmental sciences

For a reference copy of the document with all sections, see nature.com/documents/nr-reporting-summary-flat.pdf

Life sciences study design

All studies must disclose on these points even when the disclosure is negative.

Sample size	The accurate sample sizes are illustrated in the figure legends. In animal experiments of tumor growth, for individual analyses, n for each group is 6. For all other experiments, n for individual analyses is 3 or 4, with selection based on variability between estimates being sufficiently small to afford statistical significance ($p < 0.05$) between experimental groups.
Data exclusions	There were no data exclusions.
Replication	Experiments were repeated at least three times and experimental findings were reproducible.
Randomization	For all in vivo experiments, mice were randomly assigned into each group. The starting tumour burden in the treatment and control groups was similar before treatment.
Blinding	Proper blinding was applied during the data collection and analysis

Reporting for specific materials, systems and methods

We require information from authors about some types of materials, experimental systems and methods used in many studies. Here, indicate whether each material, system or method listed is relevant to your study. If you are not sure if a list item applies to your research, read the appropriate section before selecting a response.

Materials & experimental systems

n/a	Involved in the study	
☐	☒	Antibodies
☐	☒	Eukaryotic cell lines
☒	☐	Palaeontology and archaeology
☐	☒	Animals and other organisms
☒	☐	Human research participants
☒	☐	Clinical data
☒	☐	Dual use research of concern

Methods

n/a	Involved in the study	
☒	☐	ChIP-seq
☐	☒	Flow cytometry
☒	☐	MRI-based neuroimaging

Antibodies

Antibodies used	The following primary antibodies were used for FACS. They are listed as antigen first, followed by supplier (catalog number).<br>1) FITC-conjugated anti-mouse CD45, Biolegend (Cat # 103107, 1:200).<br>2) Pacific Blue™-conjugated anti-mouse/human CD11b, Biolegend (Cat # 101223, 1:100).<br>3) APC/Cyanine7-conjugated anti-mouse F4/80, Biolegend (Cat # 123117, 1:100).<br>4) PE/Cyanine7-conjugated anti-mouse CD86, Biolegend (Cat # 105013, 1:200).<br>5) PE-conjugated anti-mouse CD206, Biolegend (Cat # 141705, 1:100).<br>6) PE-conjugated anti-mouse CD3, Biolegend (Cat # 100205, 1:100).<br>7) FITC-conjugated anti-mouse CD4, Biolegend (Cat # 100406, 1:200).<br>8) APC-conjugated anti-mouse CD8a, Biolegend (Cat # 100712, 1:200).

	9) eFluor-450-conjugated anti-mouse Foxp3, Invitrogen (Cat # 48-5773-82).<br>10) Purified anti-HMGB1 Antibody, Biolegend (Cat # 651401, 1:200).<br>11) Alexa Fluor® 488 Anti-Calreticulin antibody [EPR3924], Abcam (ab196158, 1:100)<br>The following primary antibodies were used for ELISA. They are listed as antigen first, followed by supplier (catalog number).<br>1) Anti-Mouse TNF-α, Invitrogen (Cat # 50-112-9007).<br>2) Anti-Mouse IL-6, Invitrogen (Cat # 50-246-676).<br>3) Anti-Mouse IL-12, Invitrogen (Cat # EMIL12B).
Validation	1) FITC-conjugated anti-mouse CD45, Rat anti-mouse antibody, Flow cytometric analysis<br>2) Pacific Blue™-conjugated anti-mouse/human CD11b, Rat anti-mouse/human antibody, Flow cytometric analysis<br>3) APC/Cyanine7-conjugated anti-mouse F4/80, Rat anti-mouse antibody, Flow cytometric analysis<br>4) PE/Cyanine7-conjugated anti-mouse CD86, Rat anti-mouse antibody, Flow cytometric analysis<br>5) PE-conjugated anti-mouse CD206, Rat anti-mouse antibody, Flow cytometric analysis<br>6) PE-conjugated anti-mouse CD3, Rat anti-mouse antibody, Flow cytometric analysis<br>7) FITC-conjugated anti-mouse CD4, Rat anti-mouse antibody, Flow cytometric analysis<br>8) APC-conjugated anti-mouse CD8a, Rat anti-mouse antibody, Flow cytometric analysis<br>9) eFluor-450-conjugated anti-mouse Foxp3, Rat anti-mouse antibody, Flow cytometric analysis<br>10) Purified anti-HMGB1 Antibody, Mouse anti-human/mouse antibody, Western bolt/Immunocytochemistry/Immunohistochemistry<br>11) Alexa Fluor® 488 Anti-Calreticulin antibody [EPR3924], mouse anti-human antibody, Immunocytochemistry/ Immunohistochemistry<br>The following primary antibodies were used for ELISA. They are listed as antigen first, followed by supplier (catalog number).<br>1) Anti-Mouse TNF-α, Invitrogen (Cat # 50-112-9007).<br>2) Anti-Mouse IL-6, Invitrogen (Cat # 50-246-676).<br>3) Anti-Mouse IL-12, Invitrogen (Cat # EMIL12B).

Eukaryotic cell lines

Policy information about cell lines

Cell line source(s)	B16F10 cells (1101MOU-PUMC000473) and colon carcinoma MC38 (1101MOU-PUMC000523) cells were obtained from the China Infrastructure of Cell Line Resources (Chinese Academy of Medical Sciences, Beijing, China).
Authentication	Identity of the cell lines were frequently checked by their morphological features but have not been authenticated by the short tandem repeat (STR) profiling.
Mycoplasma contamination	All cell lines were tested to be mycoplasma-negative by the standard PCR method.
Commonly misidentified lines (See ICLAC register)	No commonly misidentified cell lines are used in this study.

Animals and other organisms

Policy information about studies involving animals; ARRIVE guidelines recommended for reporting animal research

Laboratory animals	6-8 week-old female mice (C57BL/6J mice) were used for all animal experiments described in this study.
Wild animals	The study did not involve wild animals.
Field-collected samples	The study did not involve samples collected from the field.
Ethics oversight	All animal experiments were performed in accordance with the guidelines approved by the Ethics Committee of Peking University.

Note that full information on the approval of the study protocol must also be provided in the manuscript.

Flow Cytometry

Plots

Confirm that:

- [x] The axis labels state the marker and fluorochrome used (e.g. CD4-FITC).
- [x] The axis scales are clearly visible. Include numbers along axes only for bottom left plot of group (a 'group' is an analysis of identical markers).
- [x] All plots are contour plots with outliers or pseudocolor plots.
- [x] A numerical value for number of cells or percentage (with statistics) is provided.

Methodology

Sample preparation	Tumours were harvested at 7 days post-treatment, treated with 1mg/mL collagenase I (Gibco, USA) for 1 h in a 37 °C water bath. Cells were filtered through nylon mesh filters with the size of 40 μm, washed with PBS and stained with fluorochrome-

	conjugated antibodies for FACS analysis.
Instrument	BD LSRFortessa
Software	BD LSRFortessa software (BD Biosciences)
Cell population abundance	The instrument counts 10,000 cells autonomously.
Gating strategy	Generally, cells were first gated on FSC-A/SSC-A. Singlet cells were usually gated using FSC-H and FSC-A.

☒ Tick this box to confirm that a figure exemplifying the gating strategy is provided in the Supplementary Information.
